# Amphetamine Injection into the Nucleus Accumbens and Electrical Stimulation of the Ventral Tegmental Area in Rats After Novelty Test—Behavioral and Neurochemical Correlates

**DOI:** 10.3390/ijms26010182

**Published:** 2024-12-28

**Authors:** Grażyna Jerzemowska, Magdalena Podlacha, Jolanta Orzeł-Gryglewska

**Affiliations:** 1 Department of Animal and Human Physiology, Faculty of Biology, University of Gdansk, 59 Wita Stwosza Str., 80-308 Gdansk, Poland; jolanta.orzel-gryglewska@ug.edu.pl; 2 Department of Molecular Biology, Faculty of Biology, University of Gdansk, 59 Wita Stwosza Str., 80-308 Gdansk, Poland; magdalena.podlacha@ug.edu.pl

**Keywords:** deep brain stimulation, nucleus accumbens shell, amphetamine, ventral tegmental area, novelty test, rats

## Abstract

Amphetamine abuse is a global health epidemic that is difficult to treat due to individual differences in response to environmental factors, including stress reactivity and anxiety levels, as well as individual neuronal differences, which may result in increased/decreased vulnerability to addiction. In the present study, we investigated whether the Wistar rats behavioral traits of high (HR) and low (LR) locomotor activity to novelty influence motivational behavior (induced feeding model; iFR by electrical stimulation of the ventral tegmental area; Es-VTA) supported by amphetamine injection into the nucleus accumbens shell (AcbSh) (HR*_Amph_*, n = 5; LR*_Amph_*, n = 5). A correlation was found between the novelty test’s locomotor activity score and the frequency threshold percentage change (*p* < 0.001, Rs = −0.867). In HR*_Amph_*, there was a shortening (−24.16%), while in LR*_Amph_*, there was a lengthening (+51.84%) of iFR latency. Immunofluorescence studies showed differential neuronal density (activity of tyrosine hydroxylase, choline acetyltransferase, and cFos protein) in the selected brain structures in HR*_Amph_* and LR*_Amph_* animals as well as in comparison to a control group (HR*_ACSF_*, n = 5; LR*_ACSF_*, n = 5). These results contribute to expanding the state of knowledge of the behavioral and neuronal propensity to take drug abuse.

## 1. Introduction

The ventral tegmental area (VTA) and the nucleus accumbens (Acb) are the most critical structures of the mesolimbic (ML) pathway, playing a pivotal role in the development of addictions. Both clusters of neurons are closely connected, and dopamine secreted by the axonal endings of the VTA and its uptake by the postsynaptic membrane of the Acb is responsible for the feeling of pleasure and satisfaction [1,2]. The Acb is a heterogeneous structure, with its various parts innervating distinct regions of the central nervous system (CNS). It is believed that the shell part of the Acb (AcbSh) sends projections mainly to the limbic system, for example, to the ventral pallidum (VP), lateral hypothalamus (LH), and VTA. In turn, part of the core (AcbC) sends projections to structures related to motor functions, such as the dorsolateral part of the VP (which transmits impulses to the subthalamic nucleus of Luys) or to the substantia nigra (SN) [3]. Acb is responsible for the organism’s motivational states and motor behaviors related to feeding, sexual, and stress functions [4]. In hungry animals and animals during eating, an increase in dopamine levels is observed in the Acb [5]. It has been found that the administration of both amphetamines [6] and opiates [7] to this structure facilitates food intake. Kelley and co-authors [8] demonstrated the crucial importance of AcbSh in the control of this response. The response to the primary stimulus in the form of food is manifested by changes in the level of extracellular dopamine in a differentiated manner in both subregions—AcbC and AcbSh. In rats, during contact with the natural unconditioned stimulus, the dopamine level increased in the AcbC without delay, regardless of the appetitive or aversive nature of the stimulus. In turn, in AcbSh, the increase in dopamine level also occurred immediately, but only in response to a stimulus with positive emotional meaning [9].

The AcbSh and AcbC also have varying degrees of responses to electrical and chemical stimulation. Stimulating different regions of the Acb with the same psychoactive substance can produce different neuronal excitation, e.g., injection of the psychomimetic drug phencyclidine, a non-competitive NMDA receptor antagonist, directly into the AcbSh causes an increase in dopaminergic transmission in the AcbSh, in contrast to the same injection directly into the AcbC, after which no increased dopaminergic transmission was observed in either the AcbSh or the AcbC [10]. Similar results were observed after the injection of cocaine, morphine, and amphetamine into different regions of the Acb [11].

Individuals differ in their behavioral and physiological responses to novel environmental stimuli in humans and animals [12,13,14,15]. Sensation-seeking is associated with high-risk behaviors, such as drug consumption, in both humans [16,17,18] and animals [19]. In animals, the main indicator differentiating behavioral types of rats in currently used experimental models is locomotor activity in a novel environment called the novelty test [19], in which the analysis of anxiety level and behavioral activity is based on computerized counting of all animal movements over a specified period. Animals characterized by high locomotor activity in a new environment are classified as high response—HR, and those characterized by low locomotor activity in a new environment are classified as low response—LR [19,20,21,22,23,24].

In many experimental systems, HR animals are more susceptible to addiction to psychoactive substances than LR animals. HR rats exhibited greater motor arousal in response to amphetamine and developed behavioral sensitization to repeated doses more quickly [19,25]. Moreover, these animals show a greater tendency to intravenously self-administer not only amphetamine [26,27,28,29] but also other addictive substances such as cocaine [30,31,32], morphine [33], or ethanol [34,35], wherein HR animals low doses of these substances produce a similar “euphoric” effect as high doses in LR animals.

Analysis of the differentiation of HR and LR animals in the context of neurochemical studies has shown higher levels of dopamine in the Acb in HR animals both at rest [31] and after cocaine [30] and corticosterone [36] injection. Also, with amphetamine self-administration, higher dopamine turnover has been observed in HR rats in the Acb and striatum and lower in the prefrontal cortex (PFC) [37]. HR animals exhibit lower expression of dopamine transporter protein (DAT) in PFC cells but show increased DAT immunoreactivity after nicotine exposure [38], as well as lower serotonin levels in the Acb, striatum, and PFC [37]. In the case of expression of the enzyme limiting dopamine synthesis—tyrosine hydroxylase (TH+), more TH+ cells were found in HR and LR animals both in the midbrain [39,40] and in the hypothalamus [41]. While many behavioral studies have examined the interactions between individual differences in stress sensitivity at rest and during self-administration of psychostimulants, no studies have directly administered psychostimulants to the AcbSh. Moreover, these studies are concerned only with assessing one marker of neuronal activity or, at most, a few selected neuronal structures. Still, there is no comprehensive “mapping” of larger brain areas in behaviorally diverse animals, and there is also a complete lack of data on changes resulting from the direct double activation of ML structures.

This study aimed to determine whether the behavioral traits of Wistar rats, differentiated in terms of locomotor activity in response to novelty, influence motivational behavior (induced feeding model; iFR by electrical stimulation of the ventral tegmental area; Es-VTA) supported by amphetamine injection into the nucleus accumbens shell (AcbSh). The next purpose of this study was to determine whether the reward system activated in this way in HR and LR rats influenced the changes in neuronal activation within the most important groups of dopaminergic (A15–A8) and cholinergic (Ch1–Ch6) cells and cFos protein.

## 2. Results

### 2.1. Behavior Analysis

The median locomotor activity score for the group used for the behavioral experiment, including all the rats (n = 30), was 3303. Rats with scores above the 4350 values were assigned to the HR group (n = 5, mean ± SD activity: 5322 ± 696) and those below the 2230 motility score to the LR group (n = 5, mean ± SD activity: 1820 ± 275). The remaining rats with an intermediary activity score between 2230 and 4350 (n = 20, mean ± SD activity: 3313 ± 212) were not included in the analyses below.

All rats’ iFR latency during Es-VTA after ACSF injection into AcbSh at individual frequencies (17.71–81.31) overlapped with the iFR latency during Es-VTA without injection into AcbSh. The percentage frequency threshold at the 20 s before and after ACSF injection oscillated within 1%.

**Figure 1:**
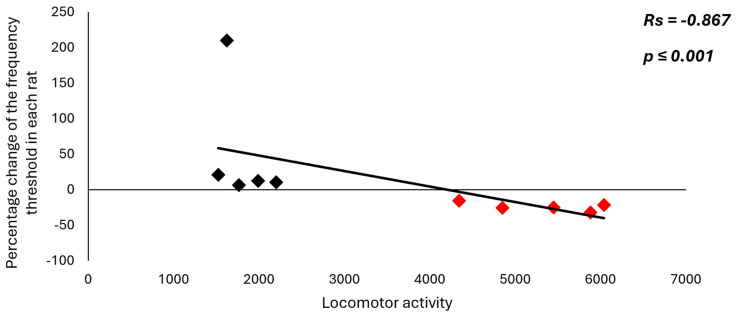
Regression analysis of percentage change in the frequency threshold in each rat (y-axis) to locomotor activity score in rats with low (LR*_Amph_*; n = 5; black rhombus) and high (HR*_Amph_*; n = 5; red rhombus) response to novelty test (x-axis). The Rs value and the corresponding *p* value show the Spearman correlation coefficient for this analysis.

**Figure 2:**
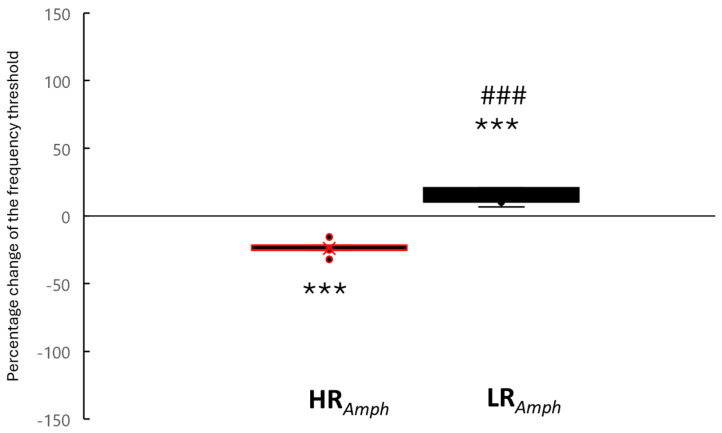
Mean (±SE) percentage change in the frequency threshold induced feeding response (iFR) during unilateral electrical VTA stimulation (Es-VTA) obtained directly after amphetamine injection (5.0 μg/0.5 μL) in the AcbSh in rats with higher locomotor activity (HR*_Amph_*; n = 5; red bar) and low locomotor activity (LR*_Amph_*; n = 5; black bar). On the y-axis, a value of 0.0% was taken as a frequency threshold behavioral iFR reaction during Es-VTA in the same rats obtained immediately after artificial cerebrospinal fluid (ACSF; a value of 0.0% on the y-axis) injection (0.5 µL) into the AcbSh (n = 10) (baseline). Explanations: Mann–Whitney U test: *p* < 0.001: *** differences from baseline for the HR rats (HR*_Amph_* vs. baseline) and the LR rats (LR*_Amph_* vs. baseline), and ### differences in a group of rats after amphetamine injection (HR*_Amph_* vs. LR*_Amph_*).

The Spearman analysis showed a negative correlation between the locomotor activity score in the novelty test in the LR (n = 5) and HR rats (n = 5) after amphetamine injection into the AcbSh and the percentage change in the frequency threshold (*p* < 0.001, Rs = −0.867) (locomotor activity with percentage change in the frequency threshold in each rat in the LR group: #1: 1524 and 20.73, #2: 1622 and 209.62, #3: 1767 and 6.54, #4: 1990 and 12.09, #5: 2200 and 10.22, and in each rat in the HR group: #1: 4344 and −15.57, #2: 4851 and −25.49, #3: 5448 and −25.10, #4: 5882 and −32.07, #5: 6042 and −21.85) (Figure 1). Moreover, comparative analysis (Mann–Whitney U test) of percentage change in the frequency in both two experimental groups also showed differences between these groups (HR*_Amph_* vs. LR*_Amph_* rats: mean % ± SE: −24.16% ± 2.68 vs. 51.84% ± 15.51; *p* < 0.001), and with the control (HR after artificial cerebrospinal fluid (ACSF) injection vs. HR*_Amph_*: *p* < 0.001 and LR after ACSF injection vs. LR*_Amph_*: *p* < 0.001) (Figure 2; percentage change in the frequency threshold for HR and LR rats after ACSF injection (control groups) has been shown as 0.0%).

**Figure 3:**
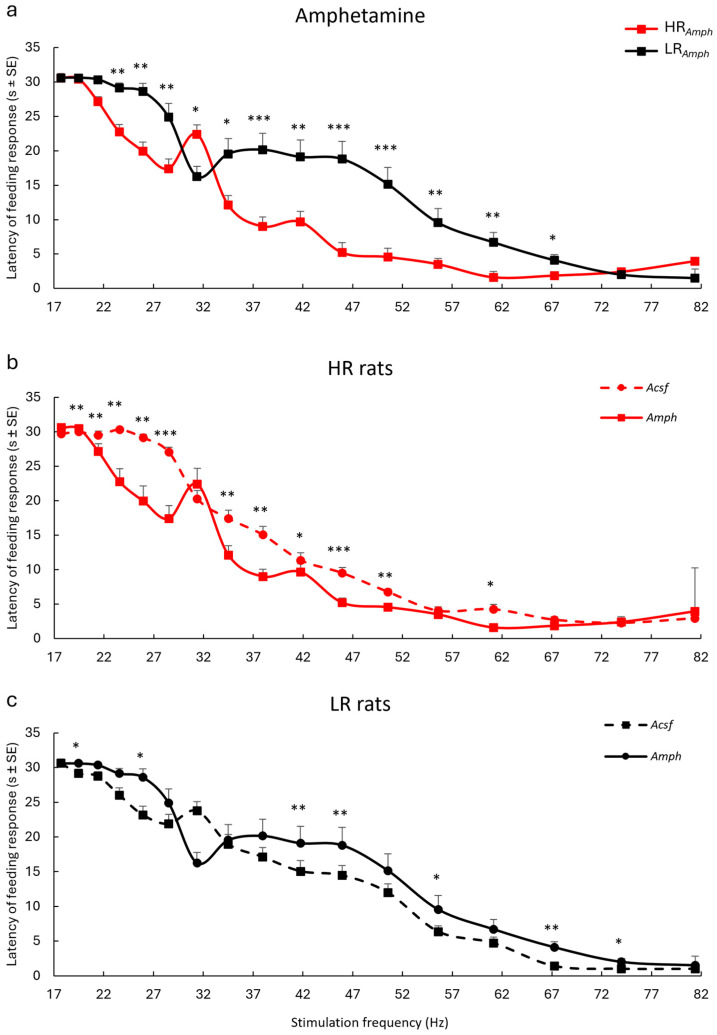
Latency of induced feeding response (iFR) by unilateral VTA electrical stimulation (Es-VTA) directly after amphetamine injection (5.0 μg/0.5 μL) into the AcbSh in the two experimental groups (HR*_Amph_*; n = 5 vs. LR*_Amph_*; n = 5) (**a**) and compared to the control which was the behavioral iFR latency obtained by Es-VTA immediately after artificial cerebrospinal fluid (ACSF) (0.5 µL) injection into the AcbSh in the same rats: HR (*Amph* vs. *Acsf*) (**b**) and LR (*Amph* vs. *Acsf*) (**c**). Explanation: Wilcoxon’s signed rank test: * *p* < 0.05, ** *p* < 0.01 and *** *p* < 0.001, differences in the iFR latency at different frequencies of Es-VTA in the experimental groups (HR*_Amph_* vs. LR*_Amph_*) and compared to the control (*Amph* vs. *Acsf*).

The latency of the iFR showed an interaction between the amphetamine injection into the AcbSh and the frequency of the stimulation current in the VTA (from 25.95 Hz to 67.25 Hz) in both experimental groups (HR*_Amph_* and LR*_Amph_* rats). It was manifested by its shortening in the HR group, especially at the eleven subjected frequencies 37.96 Hz (z = −3.494), 45.94 Hz (z = −3.510), 50.53 Hz (z = −3.341), (*p* < 0.001), 23.57 Hz (z = −2.684), 25.95 Hz (z = −2.743), 28.52 Hz (−2.746), 41.76 Hz (z = −3.046), 55.53 Hz (z = −2.751), and 61.14 Hz (z = −2.809) (*p* < 0.01) and at the 67.25 Hz (z = −2.549) and 34.51 Hz (z = −2.535) (*p* < 0.05). The exception was the frequency of 31.37 Hz, at which a longer iFR latency was observed in rats from the HR*_Amph_* group than in the LR*_Amph_* group (z = −2.542) (*p* < 0.05). Changes in response latency in both experimental groups were not observed (*p* ≥ 0.05) at the three lowest 17.71 Hz (z = 0.0), 19.48 Hz (z = −0.832) and 21.48 Hz (z = −1.944) and two highest frequencies 73.98 Hz (z = −0.508) and 81.38 Hz (z = −1.753) (Wilcoxon’s signed rank test) (Figure 3a).

Comparative analysis of experimental groups (*Amph*) with control groups (*Acsf*) in iFR latency at specific frequencies (17.71–81.38) during electrical stimulation of the VTA showed different behavioral activity during this stimulation, which corresponded to the locomotor activity of rats (HR and LR rats) (Wilcoxon’s signed rank test) (Figure 3b,c). In the case of HR animals, in the experimental group (*Amph*) as compared to the control (*Acsf*), a shortening of iFR latency was observed at most of the analyzed frequencies: 19.48 Hz (z = −3.051; *p* < 0.01), 21.48 Hz (z = −2.807; *p* < 0.01), 23.57 (z = −2.807; *p* < 0.01), 25.98 Hz (z = −3.172; *p* < 0.01), 28.52 Hz (z = −3.222, *p* < 0.001), 34.51 Hz (z = −2.751, *p* < 0.01), 37.96 Hz (z = −2.799; *p* < 0.01), 41.76 Hz (z = −2.132; *p* < 0.05), 48.94 Hz (z = −3.306; *p* < 0.001), 50.53 Hz (z = −2.970; *p* < 0.01), 61.14 Hz (z = −2.376; *p* < 0.05) (Figure 3b), while in LR animals (*Amph* vs. *Acsf*), its prolongation was observed for the following frequencies: 19.48 Hz (z = −1.998; *p* ≤ 0.05), 25.98 Hz (z = −2.483; *p* < 0.05), 41.76 Hz (z = −2.571; *p* < 0.01), 48.97 Hz (z = −2.655; *p* < 0.01), 55.58 Hz (z = −2.132; *p* < 0.01), 67.25 Hz (z = −2.814; *p* < 0.01) and 73.98 Hz (z = −2.032; *p* < 0.05) (Figure 3c).

### 2.2. Immunofluorescence Analysis

The neurochemical studies used the brains of rats that underwent behavioral analysis after amphetamine injection, forming the experimental groups (HR*_Amph_*; n = 5 and LR*_Amph_*; n = 5). To compare neuronal activity between HR/LR animals after amphetamine and ACSF injections, an additional control group was formed. This control group underwent the same procedures as the experimental group, except receiving only a unilateral ACSF injection into the AcbSh before the Es-VTA. Before the stimulation procedure, all animals in the study (n = 30) underwent a novelty test to distinguish between those with high and low locomotor activity. The median (± SD) locomotor activity score for the control group used only for the neurochemical experiment, including all the rats (n = 30), was 3319 (± 1109). Rats with scores above the 4428 values were assigned to the HR*_ACSF_* group (n = 5, mean ± SD activity: 5067 ± 181) and those below the 2210 motility score to the LR*_ACSF_* group (n = 5, mean ± SD activity: 1332 ± 185). The remaining rats with an intermediary activity score between 2210 and 4428 (n = 20, mean ± SD activity: 3277 ± 162) were not included in the analyses below.

#### 2.2.1. Density of Tyrosine Hydroxylase Cells (TH+)

**Figure 4:**
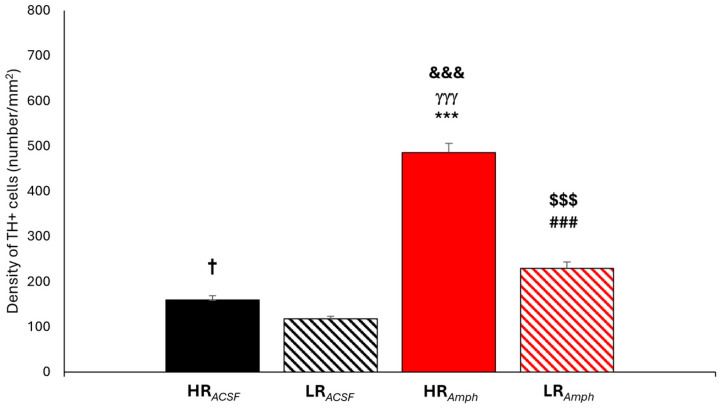
Mean (+SE) density of tyrosine hydroxylase positive cells (TH+ cells) (number/1 mm^2^) in total all analyzed dopaminergic groups (A15–A8 counted together) in animals from the experimental group (HR*_Amph_*; n = 5; red bar, and LR*_Amph_*; n = 5; red bar with stripes) and the control group (HR*_ACSF_*; n = 5; black bar, and LR*_ACSF_*; n = 5; black bar with stripes). Explanations: Wilcoxon’s signed rank test: *** *p* < 0.001, differences between the HR*_Amph_* and HR*_ACSF_*; ### *p* < 0.001, differences between the LR*_Amph_* and LR*_ACSF_*; γγγ *p* < 0.001, differences within the experimental group: HR*_Amph_* vs. LR*_Amph_* and † *p* < 0.05, differences within the control group: HR*_ACSF_* vs. LR*_ACSF_*; &&& *p* < 0.001, differences between the HR*_Amph_* and LR*_ACSF_*; $$$ *p* < 0.001, differences between the HR*_ACSF_* and LR*_Amph_*.

The statistical analysis of the TH+ cell densities within the hypothalamus (dopaminergic cell groups A15–A11) and the midbrain (dopaminergic cell groups A10–8) (Figure 4) counted together revealed that, following amphetamine injection, both behavioral groups (HR*_Amph_* and LR*_Amph_*) exhibited a higher density of TH+ cells as compared to after ACSF injection (HR*_ACSF_* and LR*_ACSF_*) (HR*_Amph_* vs. HR*_ACSF_*: z = −11.932; *p* < 0.001) (LR*_Amph_* vs. LR*_ACSF_*: z = −7.298; *p* < 0.001) (Wilcoxon’s signed rank test). Moreover, there is a difference in the density of TH+ cells in the A15–A8 dopaminergic groups analyzed together within both experimental groups (*ACSF* and *Amph* groups), where a higher density of TH+ cells was found in HR animals as compared to LR animals (HR*_ACSF_* vs. LR*_ACSF_*: z = −2.514; *p* < 0.05) (HR*_Amph_* vs. LR*_Amph_*: z = −9.516; *p* < 0.001). Additionally, there are differences in the density of TH+ between the groups HR*_Amph_* vs. LR*_ACSF_* (z = 12.162; *p* < 0.001) and HR*_ACSF_* vs. LR*_Amph_* (z = −3.401; *p* < 0.001) (Figure 4).

**Figure 5:**
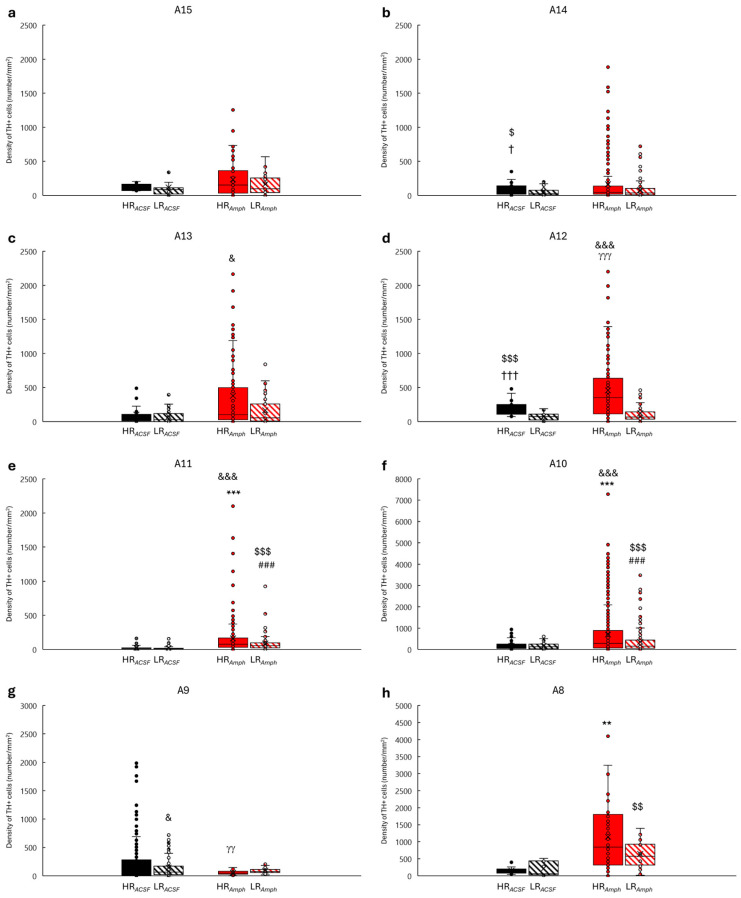
Density of TH+ cell (number/1 mm^2^) in the main dopaminergic groups A15 (**a**), A14 (**b**), A13 (**c**), A12 (**d**), A11 (**e**), A10 (**f**), A9 (**g**), and A8 (**h**) in rats after novelty test from the experimental (HR*_Amph_*; n = 5 and LR*_Amph_*; n = 5) and control groups (HR*_ACSF_*; n = 5 and LR*_ACSF_*; n = 5). Explanations: *** *p* < 0.001 and ** *p* < 0.01, differences between the HR*_Amph_* and HR*_ACSF_*; ### *p* < 0.001, differences between the LR*_Amph_* and LR*_ACSF_*; γγγ *p* < 0.001 and γγ *p* < 0.01, differences between the HR*_Amph_* and LR*_Amph_*; ††† *p* < 0.001 and † *p* < 0.05, differences between the HR*_ACSF_* and LR*_ACSF_*; &&& *p* < 0.001 and & *p* < 0.05, differences between the HR*_Amph_* and LR*_ACSF_*; $$$ *p* < 0.001, $$ *p* < 0.01 and $ *p* < 0.05, differences between the HR*_ACSF_* and LR*_Amph_* (Wilcoxon’s signed rank test).

Analysis of individual dopaminergic cell groups showed that in the hypothalamus, only in the A11 cell group was a higher density of TH+ cells in the HR animals after amphetamine injection than in the HR animals after ACSF injection (HR*_Amph_* vs. HR*_ACSF_*: z = −6.366; *p* < 0.001) (Figure 5e) (Wilcoxon’s signed rank test). A similar increasing trend was observed in LR animals (LR*_Amph_* vs. LR*_ACSF_*: z = −6.348; *p* < 0.001) (Figure 5e). In the midbrain, only in the VTA (dopaminergic cell group A10) and in the retrorubral field (RRF) (dopaminergic cell group A8), a significant increase in TH+ cell density was observed in HR animals after amphetamine injection (A10: HR*_Amph_* vs. HR*_ACSF_*: z = −3.584; *p* < 0.001) (Figure 5f) (A8: HR*_Amph_* vs. HR*_ACSF_*: z = −2.817; *p* < 0.01) (Figure 5h). In LR animals (*Amph* group vs. *ACSF* group), a significant increase in TH+ density was found only in the VTA in the A10 dopaminergic cell group (LR*_Amph_* vs. LR*_ACSF_*: z = −4.419; *p* < 0.001) (Figure 5f).

Furthermore, in both the hypothalamus and midbrain, variation was observed within both behavioral groups (HR vs. LR) after both ACSF injection (HR*_ACSF_* vs. LR*_ACSF_*) and amphetamine injection (HR*_Amph_* vs. LR*_Amph_*). In the hypothalamus, this was primarily the case for cells from A14 (HR*_ACSF_* vs. LR*_ACSF_*: z = −2.083; *p* < 0.05) (Figure 5b) and A12 (HR*_ACSF_* vs. LR*_ACSF_*: z = −4.057; *p* < 0.001) (HR*_Amph_* vs. LR*_Amph_*: z = −4.182; *p* < 0.001) (Figure 5d), and in the midbrain of the A9 cell group in the SN (HR*_Amph_* vs. LR*_Amph_*: z = −3.60; *p* < 0.01) (Figure 5g).

**Figure 6:**
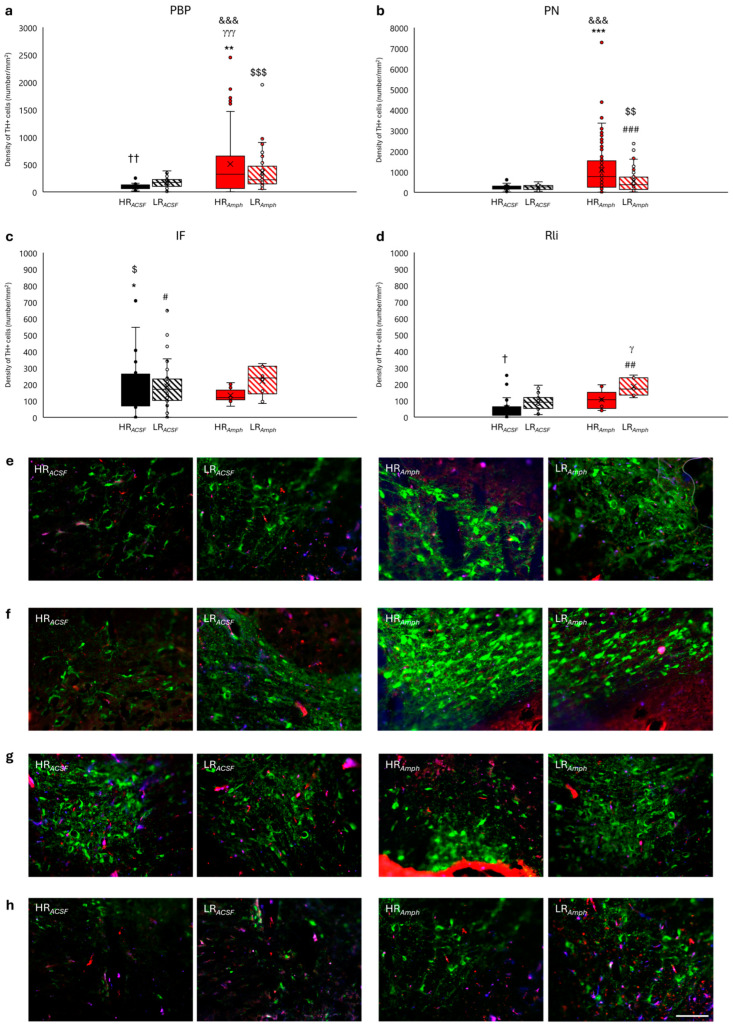
Density of TH+ cell (number/1 mm^2^) in the selected paired nuclei of the ventral tegmental area (VTA): parabrachial pigmentosus (PBP) (graph (**a**), photos (**e**)) and paranigral (PN) (graph (**b**), photos (**f**)) and in the unpaired nuclei of the VTA arranged in the midline axis: interfascicular (IF) (graph (**c**), photos (**g**)) and raphe linear, pars rostral (Rli) (graph (**d**), photos (**h**)) which constitute a group of A10 dopaminergic cells in brain rats after novelty test from the experimental (HR*_Amph_*; n = 5 and LR*_Amph_*; n = 5) and control groups (HR*_ACSF_*; n = 5 and LR*_ACSF_*; n = 5). The microphotos ((**e**–**h**) panels) show TH+ (green signal), cFos+ (red signal), and DAPI (blue signal) labeled neurons. Scale bar = 100 µm: white line: right lower corner of the last photo, panel h (fluorescent microscope PrimoStar from Carl Zeiss MicroImaging GmbH, Göttingen, Germany; picture definition 1024 × 1024 points; computer program Axio Vision Rel4.8 from Carl Zeiss Imaging System; magnification 20 × 10). Explanations for the graphs: *** *p* < 0.001, ** *p* < 0.01 and * *p* < 0.05, differences between the HR*_Amph_* and HR*_ACSF_*; ### *p* < 0.001, ## *p* < 0.01 and # *p* < 0.05, differences between the LR*_Amph_* and LR*_ACSF_*; γγγ *p* < 0.001 and γ *p* < 0.05, differences between the HR*_Amph_* and LR*_Amph_*; †† *p* < 0.01 and † *p* < 0.05, differences between the HR*_ACSF_* and LR*_ACSF_*; &&& *p* < 0.001, differences between the HR*_Amph_* and LR*_ACSF_* and $$$ *p* < 0.001, $$ *p* < 0.01 and $ *p* < 0.05, differences between the HR*_ACSF_* and LR*_Amph_* (Wilcoxon’s signed rank test).

Analysis of individual VTA structures (cell group A10) (Figure 6a–h) showed an increase in TH+ cell density in the *Amph* group compared to the *ACSF* group in both evenly stacked nuclei, such as parabrachial pigmented (PBP) (HR*_Amph_* vs. HR*_ACSF_*: z = −3.162; *p* < 0.01) (Figure 6a,e) and paranigral (PN) nuclei (HR*_Amph_* vs. HR*_ACSF_*: z = −3.786; *p* < 0.001) (Figure 6b,f), and PN (LR*_Amph_* vs. LR*_ACSF_*: z = −3.300; *p* < 0.001) (Figure 6b,f), as well as in the midline nuclei as interfascicularis (IF) (HR*_Amph_* vs. HR*_ACSF_*: z = −2.497; *p* < 0.05) (LR*_Amph_* vs. LR*_ACSF_*: z = −2.380; *p* < 0.05) (Figure 6c,g) and rostralis (Rli) nuclei (LR*_Amph_* vs. LR*_ACSF_*: z = −2.666; *p* < 0.01) (Figure 6d,h). In addition, the variation in TH+ cell density within the behavioral groups (HR vs. LR) after both ACSF (HR*_ACSF_* vs. LR*_ACSF_*) and amphetamine (HR*_Amph_* vs. LR*_Amph_*) injection was also maintained in these structures. A higher density of TH+ cells was observed in HR animals after amphetamine injection in the PBP nucleus (HR*_Amph_* vs. LR*_Amph_*: z = −3.936; *p* < 0.001) (Figure 6a,e) and in LR animals after ACSF injection both in PBP (HR*_ACSF_* vs. LR*_ACSF_*: z = −2.578; *p* < 0.01) (Figure 6a,e) and in Rli (HR*_ACSF_* vs. LR*_ACSF_*: z = −2.299; *p* < 0.05) (Figure 6d,h), as well as in LR rats after amphetamine injection in Rli (HR*_Amph_* vs. LR*_Amph_*: z = −2.366; *p* < 0.05) (Figure 6d,h).

**Figure 7:**
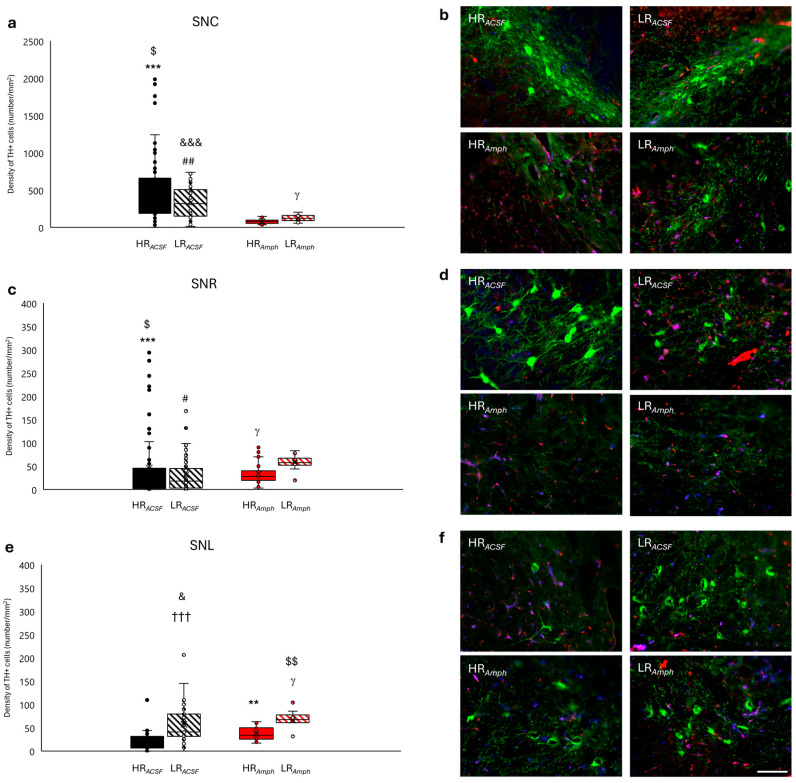
Density of TH+ cell (number/1 mm^2^) in the structures of the substantia nigra: pars compacta (SNC) (graph (**a**), photos (**b**)), reticulata (SNR) (graph (**c**), photos (**d**)) and lateralis (SNL) (graph (**e**), photos (**f**)) which constitute a group of A9 dopaminergic cells in brain rats after novelty test from the experimental (HR*_Amph_*; n = 5 and LR*_Amph_*; n = 5) and control groups (HR*_ACSF_*; n = 5 and LR*_ACSF_*; n = 5). The microphotos ((**b**), (**d**), (**f**) panels) show TH+ (green signal), cFos+ (red signal), and DAPI (blue signal) labeled neurons. Scale bar = 100 µm: white line: right lower corner of the last photo, panel **f** (fluorescent microscope PrimoStar from Carl Zeiss MicroImaging GmbH, Göttingen, Germany; picture definition 1024 × 1024 points; computer program Axio Vision Rel4.8 from Carl Zeiss Imaging System; magnification 20 × 10). Explanations for the graphs: *** *p* < 0.001 and ** *p* < 0.01, differences between the HR*_Amph_* and HR*_ACSF_*; ## *p* < 0.01 and # *p* < 0.05, differences between the LR*_Amph_* and LR*_ACSF_*; γ *p* < 0.05, differences between the HR*_Amph_* and LR*_Amph_*; ††† *p* < 0.001, differences between the HR*_ACSF_* and LR*_ACSF_*; &&& *p* < 0.001 and & *p* < 0.05, differences between the HR*_Amph_* and LR*_ACSF_* and $$ *p* < 0.01 and $ *p* < 0.05, differences between the HR*_ACSF_* and LR*_Amph_* (Wilcoxon’s signed rank test).

In the SN, the distribution of TH+ cell density was unequal in both HR and LR animals after amphetamine injection (HR*_Amph_* and LR*_Amph_*) compared to the control (HR*_ACSF_* and LR*_ACSF_*) in its respective parts: compact (SNC), reticular (SNR) and lateral (SNL) (Figure 7a–f). In the *Amph* group, a decrease in TH+ cell density was observed compared to the *ACSF* group in HR animals in both parts of SN: SNC (HR*_Amph_* vs. HR*_ACSF_*: z = −3.888; *p* < 0.001) (Figure 7a,b) and in SNR (HR*_Amph_* vs. HR*_ACSF_*: z = −3.393; *p* < 0.001) (Figure 7c,d). A similar trend of TH+ cell density was observed as well in LR animals in the SNC (LR*_Amph_* vs. LR*_ACSF_*: z = −2.589; *p* < 0.01) (Figure 7a,b). In contrast, higher TH+ density after amphetamine injection (*Amph* group) compared to controls (*ACSF* group) was only observed in LR animals in the SNR (LR*_Amph_* vs. LR*_ACSF_*: z = −1.960; *p* < 0.05) (Figure 7c,d) and HR animals in the SNL (HR*_Amph_* vs. HR*_ACSF_*: z = −2.182; *p* < 0.05) (Figure 7e,f). When comparing behavioral groups within the experimental (HR*_Amph_* vs. LR*_Amph_*) and control (HR*_ACSF_* vs. LR*_ACSF_*) groups, there was a higher TH+ cell density in LR animals at the SNC (z = −2.275; *p* < 0. 05), SNR (z = −2.521; *p* < 0.05) and SNL (z = −2.192; *p* < 0.05) in the *Amph* group (HR*_Amph_* vs. LR*_Amph_*) (Figure 7a–f) and SNL (z = −4.633; *p* < 0.001) in the *ACSF* group (HR*_ACSF_* vs. LR*_ACSF_*) (Figure 7e,f).

Moreover, Figure 5a–h, Figure 6a–d and Figure 7a,c,e additionally present the differences between the experimental and control groups (HR*_Amph_* vs. LR*_ACSF_* and HR*_ACSF_* vs. LR*_Amph_*) (Wilcoxon’s signed rank test).

#### 2.2.2. Density of Choline Acetyltransferase Cells (ChAT+)

**Figure 8:**
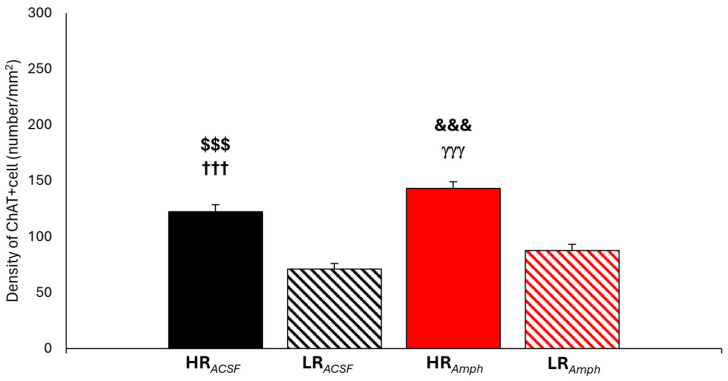
Mean (+SE) density of choline acetyltransferase positive cells (ChAT+ cells) (number/1 mm^2^) in total all analyzed cholinergic groups (Ch1–Ch6 counted together) in animals from the experimental group (HR*_Amph_*; n = 5; red bar, and LR*_Amph_*; n = 5; red bar with stripes) and the control group (HR*_ACSF_*; n = 5; black bar, and LR*_ACSF_*; n = 5; black bar with stripes). Explanations: Wilcoxon’s signed rank test: γγγ *p* < 0.001, differences within the experimental group: HR*_Amph_* vs. LR*_Amph_* and ††† *p* < 0.01, differences within the control group: HR*_ACSF_* vs. LR*_ACSF_*; &&& *p* < 0.001, differences between the HR*_Amph_* and LR*_ACSF_*; $$$ *p* < 0.001, differences between the HR*_ACSF_* and LR*_Amph_*.

Statistical analysis of the density in all together counted groups of cholinergic cells Ch1–Ch6 showing ChAT+ activity (Figure 8) showed their differentiation only within the locomotor activity of both groups: experimental (HR*_Amph_* vs. LR*_Amph_*) and control (HR*_ACSF_* vs. LR*_ACSF_*). Higher level density of ChAT+ was observed in HR animals ( HR*_Amph_* vs. LR*_Amph_*: z = −5.115; *p* < 0.001; HR*_ACSF_* vs. LR*_ACSF_*: z = −5.590; *p* < 0.001) (Wilcoxon signed-rank test) (Figure 8). Unfortunately, there was no difference in ChAT+ cell level density between the experimental and control groups (HR*_Amph_* vs. HR*_ACSF_*; z = −0.316; *p* > 0.05 and LR*_Amph_* vs. LR*_ACSF_*; z = −1.914; *p* > 0.05) (Wilcoxon’s signed rank test) (Figure 8).

**Figure 9:**
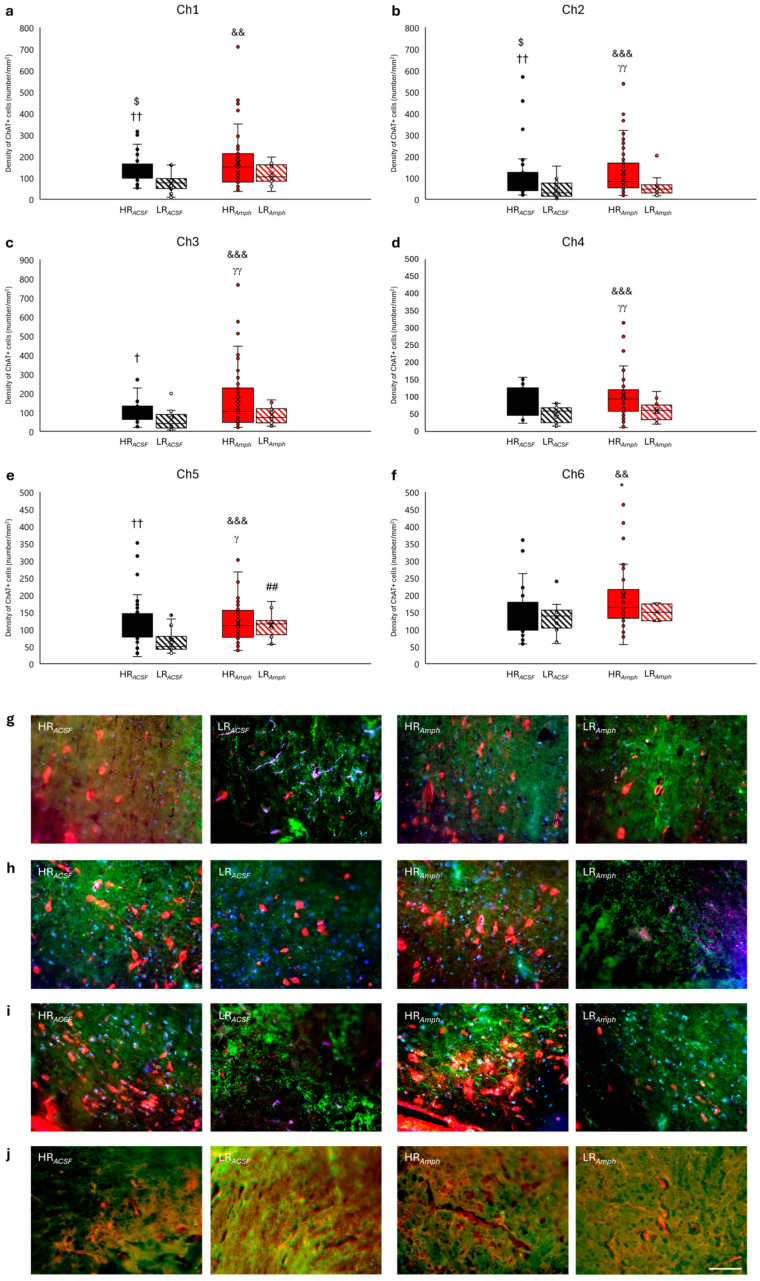
Density of ChAT+ cell (number/1 mm^2^) in the main cholinergic groups Ch1 (graph (**a**), photos (**g**)), Ch2 (graph (**b**), photos (**h**)), Cg3 (graph (**c**), photos (**i**)), Ch4 (graph (**d**)), Ch5 (graph (**e**), photos (**j**)) and Ch6 (graph (**f**)) in rats after novelty test from the experimental (HR*_Amph_*; n = 5 and LR*_Amph_*; n = 5) and control groups (HR*_ACSF_*; n = 5 and LR*_ACSF_*; n = 5). The representative microphotos ((**g**–**j**) panels) show ChAT+ (red signal), TH+ (green signal), and DAPI (blue signal) labeled neurons. Scale bar = 100 µm: white line: right lower corner of the last photo, panel (**j**) (fluorescent microscope PrimoStar from Carl Zeiss MicroImaging GmbH, Göttingen, Germany; picture definition 1024 × 1024 points; computer program Axio Vision Rel4.8 from Carl Zeiss Imaging System; magnification 20 × 10). Explanations for the graphs: * *p* < 0.05, differences between the HR*_Amph_* and HR*_ACSF_*; ## *p* < 0.01, differences between the LR*_Amph_* and LR*_ACSF_*; γγ *p* < 0.01 and γ *p* < 0.05, differences between the HR*_Amph_* and LR*_Amph_*; †† *p* < 0.01 and † *p* < 0.05, differences between the HR*_ACSF_* and LR*_ACSF_*; &&& *p* < 0.001 and && *p* < 0.01, differences between the HR*_Amph_* and LR*_ACSF_* and $ *p* < 0.05, differences between the HR*_ACSF_* and LR*_Amph_* (Wilcoxon’s signed rank test).

Separately analysis for each Ch1–Ch6 cholinergic cell group was also observed; the most significant differentiation in their density was also observed mainly in animals divided according to locomotor activity: in experimental (HR*_Amph_* vs. LR*_Amph_*) and control (HR*_ACSF_* vs. LR*_ACSF_*) (Figure 9). In the control group, HR*_ACSF_* animals in comparison to LR*_ACSF_* animals showed a higher density of ChAT+ in the areas of Ch1 cells located in the medial septum (MS) (z = −2.809; *p* < 0.01) (Figure 9a,g), and also in the areas of the Ch2–Ch3 cells located in the nucleus of the horizontal (HDB) and ventral (VDB) limb of the diagonal band (Ch2: z = −2.914; *p* < 0.01, and Ch3: z = −1.982; *p* < 0.05) (Figure 9b,c,h,i), and in Ch5 located in the pedunculopontine tegmental nucleus (PPN) (z = −2.866; *p* < 0.01) (Figure 9e,j). In the experimental group (HR*_Amph_* vs. LR*_Amph_*), a higher density of ChAT+ was found in the area of the Ch2 cells (z = −2.809; *p* < 0.01) (Figure 9b,h), Ch3 (z = −2.783; *p* < 0.01) (Figure 9c,i), Ch4 (z = −2.919; *p* < 0.01) (Figure 9d) and Ch5 (z = −2.166; *p* < 0.05) (Figure 9e,j).

In HR animals, after amphetamine injection (experimental group), there was no significant difference in the density of ChAT+ cells within the most important groups of cholinergic cells of the Ch1–Ch5 type as compared to HR animals after ACSF injection (control group), except for the group of Ch6 cells located within the laterodorsal tegmental nucleus (LDT) (HR*_Amph_* vs. HR*_ACSF_*: z = −2.527; *p* < 0.05) (Figure 9a–j). In LR animals after amphetamine injection (experimental group), compared to LR animals after ACSF injection (control group), a higher density of ChAT+ cell was observed only in the Ch5 (PPN) group (LR*_Amph_* vs. LR*_ACSF_*: z = −2.982; *p* < 0.01) (Figure 9e–j).

In addition, Figure 9a–f and Figure 9g,h additionally present the differences between the experimental and control groups (HR*_Amph_* vs. LR*_ACSF_* and HR*_ACSF_* vs. LR*_Amph_*) (Wilcoxon’s signed rank test).

#### 2.2.3. Density of Nuclei with cFos Protein Expression (cFos+)

Microscopic analysis of cFos+ nuclei was performed mainly in the forebrain and midbrain. Selected structures (57 structures) were grouped anatomically and functionally to illustrate better the comparisons between the experimental groups (HR*_Amph_*, n = 5; LR*_Amph_*, n = 5) and the control groups (HR*_ACSF_*, n = 5; LR*_ACSF_*, n = 5). The results were presented as mean (± SE) (Wilcoxon’s signed rank test) in eight tables and concerned selected structures of the cortex (Table 1), septum (Table 2), hippocampus (Table 3), amygdala (Table 4), thalamus (Table 5), supra- and hypothalamus (Table 6), limbic structures of the midbrain (Table 7) and the remaining structures classified as extrapyramidal structures and ventral striatum (Table 8).

In selected structures of the limbic cerebral cortex, HR animals showed increased cFos+ protein density after amphetamine injection (HR*_Amph_* vs. HR*_ACSF_*) in the cingulate cortex, areas of CG1 (z = −3.110; *p* < 0.01) and CG2 (z = −3.180; *p* < 0.001), and retrosplenial granular (RSG) and agranular cortex (RSA) (z = −2.666; *p* < 0.01) except the perirhinal cortex (PRh) (z = −0.507; *p* > 0.05). In the case of LR animals (LR*_Amph_* vs. LR*_ACSF_*), this relationship was also visible but to a lesser extent in CG2 (z = −2.722; *p* < 0.01), PRh (z = −2.380; *p* < 0.05), RSA (z = −2.547; *p* < 0.05) and RSG (z = −2.666; *p* < 0.01), except CG1 (z = −1.917; *p* > 0.05). Moreover, in the experimental group, a higher concentration of cFos+ protein was found in HR animals compared to LR animals (HR*_Amph_* vs. LR*_Amph_*) in CG1 (z = −2.481; *p* < 0.05), RSA (z = −2.666; *p* < 0.01) and RSG (z = −2.310; *p* < 0.05) and the control group (HR*_ACSF_* vs. LR*_ACSF_*) in PRh (z = −2.824; *p* < 0.01), RSA (z = −2.971; *p* < 0.01) and RSG (z = −2.551; *p* < 0.05) (Table 1).

**Table 1:** Density of cFos+ cell nuclei (number/1 mm^2^) (mean ±SE) in select limbic structures of the cortex: cingulate cortex, areas of 1 (CG1) and 2 (CG2), peripheral cortex (PRh), and retrosplenial granular (RSG) and agranular cortex (RSA) in the brains of rats after novelty test from the experimental (HR*_Amph_*; n = 5; and LR*_Amph_*; n = 5) and control (HR*_ACSF_*; n = 5; and LR*_ACSF_*; n = 5) groups.

Cortex	Control Groups		Experimental Groups	
	HR*_ACSF_*	LR*_ACSF_*	HR*_Amph_*	LR*_Amph_*
CG1	13.38 ± 3.36	20.39 ± 4.76	72.49 ± 7.89 ^**,γ,&&&^	33.03 ± 6.45 ^$^
CG2	18.63 ± 8.54	16.97 ± 4.52	68.75 ± 18.94 ^***,&&^	64.97 ± 10.91 ^##,$$^
PRh	118.04 ± 41.55 ^††^	17.05 ± 5.15	135.74 ± 37.86 ^&^	103.14 ± 23.34 ^#^
RSA	48.82 ± 12.11 ^††^	12.28 ± 2.16	124.92 ± 12.28 ^**,γγ,&&^	53.11 ± 14.05 ^#^
RSG	24.55 ± 3.40 ^†^	5.77 ± 1.73	116.64 ± 5.26 ^**,γ,&&^	59.62 ± 14.86 ^##^

Explanations: *** *p* < 0.001 and ** *p* < 0.01, differences between the HR*_Amph_* and HR*_ACSF_*; ## *p* < 0.01 and # *p* < 0.05, differences between the LR*_Amph_* and LR*_ACSF_*; γγ *p* < 0.01 and γ *p* < 0.05, differences between the HR*_Amph_* and LR*_Amph_*; †† *p* < 0.01 and † *p* < 0.05, differences between the HR*_ACSF_* and LR*_ACSF_*; &&& *p* < 0.001, && *p* < 0.01 and & *p* < 0.05, differences between the HR*_Amph_* and LR*_ACSF_*; $$ *p* < 0.01 and $ *p* < 0.05, differences between the HR*_ACSF_* and LR*_Amph_* (Wilcoxon’s signed rank test).

In the case of the septum (Table 2), more cFos+ protein was detected in animals from both experimental groups (HR*_Amph_* and LR*_Amph_*) compared to the control (HR*_ACSF_* and LR*_ACSF_*) in most of the analyzed structures: lateral septum, parts dorsal (LSD), intermediate (LSI) and ventral (LSV) (HR*_Amph_* vs. HR*_ACSF_*: z = −2.803; *p* < 001 and LR*_Amph_* vs. LR*_ACSF_*: z = −4.167, *p* < 001), and MS (HR*_Amph_* vs. HR*_ACSF_*: z = −2.201; *p* < 0.05 and LR*_Amph_* vs. LR*_ACSF_*: z = −3.823; *p* < 0.001), HDB (LR*_Amph_* vs. LR*_ACSF_*: z = −1.955; *p* < 0.05) and VDB nuclei (HR*_Amph_* vs. HR*_ACSF_*: z = −2.521; *p* < 0.05 and LR*_Amph_* vs. LR*_ACSF_*: z = −3.408; *p* < 0.001).

**Table 2:** Density of cFos+ cell nuclei (number/1 mm^2^) (mean ±SE) in the septum: lateral septum dorsal (LSD), intermediate (LSI) and ventral (LSV) part, medial septum nucleus (MS) and nuclei of ventral (VDB) and horizontal (HDB) limb of the diagonal band in the brains of rats after novelty test from the experimental (HR*_Amph_*; n = 5; and LR*_Amph_*; n = 5) and control (HR*_ACSF_*; n = 5; and LR*_ACSF_*; n = 5) groups.

Septum	Control Groups		Experimental Groups	
	HR*_ACSF_*	LR*_ACSF_*	HR*_Amph_*	LR*_Amph_*
LSD	21.52 ± 4.61	13.69 ± 1.38	109.12 ± 18.49 ^**,&&^	103.46 ± 8.01 ^###,$$$^
LSI	14.56 ± 4.11	7.06 ± 0.83	53.80 ± 13.67 ^**,&&^	40.05 ± 2.33 ^###,$$^
LSV	17.52 ± 6.92	7.84 ± 0.88	86.51 ± 15.41 ^**,γγ,&&^	68.15 ± 5.01 ^###,$$$^
MS	16.94 ± 3.84	14.36 ± 2.23	67.98 ± 16.75 ^*,γ,&^	105.86 ± 7.26 ^###,$$^
VDB	25.87 ± 9.76	15.11 ± 2.07	64.04 ± 14.01 ^*,γ,&&^	87.15 ± 12.39 ^###,$^
HDB	44.10 ± 9.23 ^†^	14.75 ± 2.34	57.06 ± 11.03	32.02 ± 6.50 ^#^

Explanations: ** *p* < 0.01 and * *p* < 0.05, differences between the HR*_Amph_* and HR*_ACSF_*; ### *p* < 0.001 and # *p* < 0.05, differences between the LR*_Amph_* and LR*_ACSF_*; γγ *p* < 0.01 and γ *p* < 0.05, differences between the HR*_Amph_* and LR*_Amph_*; † *p* < 0.05, differences between the HR*_ACSF_* and LR*_ACSF_*; && *p* < 0.01 and & *p* < 0.05, differences between the HR*_Amph_* and LR*_ACSF_*; $$$ *p* < 0.001, $$ *p* < 0.01 and $ *p* < 0.05, differences between the HR*_ACSF_* and LR*_Amph_* (Wilcoxon’s signed rank test).

In the hippocampus, no differences in c-Fos+ protein density were found in animals after amphetamine administration (HR*_Amph_* and LR*_Amph_*) compared to controls (HR*_ACSF_* and LR*_ACSF_*) (*p* > 0.05) (Table 3).

**Table 3:** Density of cFos+ cell nuclei (number/1 mm^2^) (mean ±SE) in the hippocampus: CA1–CA3 fields and dentate gyrus (DG) in the brains of rats after novelty test from the experimental (HR*_Amph_*; n = 5; and LR*_Amph_*; n = 5) and control (HR*_ACSF_*; n = 5; and LR*_ACSF_*; n = 5) groups.

Hippocampus	Control Groups		Experimental Groups	
	HR*_ACSF_*	LR*_ACSF_*	HR*_Amph_*	LR*_Amph_*
CA1	59.05 ± 15.91	14.34 ± 7.18	128.99 ± 28.91	90.56 ± 37.84
CA2	50.04 ± 10.69 ^†^	21.53 ± 6.85	116.90 ± 18.47	112.46 ±24.95
CA3	54.25 ± 5.23 ^†^	25.05 ± 9.54	136.11 ± 29.79	82.52 ± 28.36
DG	17.24 ± 5.06	7.10 ± 6.53	67.47 ± 0.69	54.50 ± 2.90

Explanations: † *p* < 0.05, differences between the HR*_ACSF_* and LR*_ACSF_* (Wilcoxon’s signed rank test).

In selected amygdala nuclei (Table 4), higher c-Fos+ density after amphetamine injection was observed in LR animals in all analyzed structures: the antero-cortical nucleus (ACo) (LR*_Amph_* vs. LR*_ACSF_*: z = −3.233; *p* < 0.001), the anterior part of the basolateral nucleus (BLA) and bed nucleus of the stria terminalis (BST) (LR*_Amph_* vs. LR*_ACSF_*: z = −4.703; *p* > 0.001), the central (Ce) (LR*_Amph_* vs. LR*_ACSF_*: z = −3.989; *p* < 0.001) and medial nuclei of the amygdala (Me) (LR*_Amph_* vs. LR*_ACSF_*: z = −3.636; *p* < 0.001). In HR animals, higher cFos+ density was observed only in BST (z = −3.059; *p* < 0.01), Ce (z = −2.605; *p* < 0.01), and Me (z = −2.521; *p* < 0.01).

**Table 4:** Density of cFos+ cell nuclei (number/1 mm^2^) (mean ±SE) in the amygdala nuclei: antero-cortical (ACo) and basolateral (BLA) nuclei, bed nucleus of the stria terminalis (BST) central (Ce) and medial (Me) nuclei in the brains of rats after novelty test from the experimental (HR*_Amph_*; n = 5; and LR*_Amph_*; n = 5) and control (HR*_ACSF_*; n = 5; and LR*_ACSF_*; n = 5) groups.

Amygdala	Control Groups		Experimental Groups	
	HR*_ACSF_*	LR*_ACSF_*	HR*_Amph_*	LR*_Amph_*
ACo	90.35 ± 17.79 ^†^	21.39 ± 4.31	153.44 ± 28.30 ^&&^	105.94 ± 4.44 ^###^
BLA	55.26 ± 14.61 ^†^	11.37 ± 2.77	101.42 ± 16.50 ^&&^	71.87 ±4.95 ^###^
BST	13.32 ± 2.86 ^†^	10.06 ± 2.07	137.58 ± 14.60 ^**,γγ,&&^	78.52 ± 4.35 ^###,$$^
Ce	33.08 ± 6.03 ^††^	10.28 ± 2.71	87.01 ± 12.00 ^**,γ,&&^	56.58 ± 3.81 ^###,$$^
Me	33.07 ± 6.05	17.45 ± 4.86	202.52 ± 38.56 ^*,&^	135.96 ± 18.14 ^###,$$$^

Explanations: ** *p* < 0.01 and * *p* < 0.05, differences between the HR*_Amph_* and HR*_ACSF_*; ### *p* < 0.001, differences between the LR*_Amph_* and LR*_ACSF_*; γγ *p* < 0.01 and γ *p* < 0.05, differences between the HR*_Amph_* and LR*_Amph_*; †† *p* < 0.01 and † *p* < 0.05, differences between the HR*_ACSF_* and LR*_ACSF_*; && *p* < 0.01 and & *p* < 0.05, differences between the HR*_Amph_* and LR*_ACSF_*; $$$ *p* < 0.001 and $$ *p* < 0.01, differences between the HR*_ACSF_* and LR*_Amph_* (Wilcoxon’s signed rank test).

In the thalamus (Table 5), in the HR*_Amph_* and LR*_Amph_* animals as compared to the HR*_ACSF_* and LR*_ACSF_* animals, there was significantly more cFos+ protein in the limbic nuclei of the thalamus: the anterodorsal (AD) (HR: z = −2.366; *p* < 0.05) (LR: z = −2.666; *p* < 0.01), anteromedial (AM) (HR: z = −2.197; *p* < 0.05) (LR: z = −2.521; *p* < 0.05), anteroventral (AV) (HR: z = −3.180; *p* < 0.001) (LR: z = −3.516; *p* < 0.001), mediodorsal (MD) nuclei (HR: z = −2.934; *p* < 0.01) (LR: z = −2.472; *p* < 0.05), and lateral (LHb) (LR: z = −2.521; *p* < 0.05) and medial habenula (MHb) (HR: z = −2.521; *p* < 0.05) (LR: z = −3.296; *p* < 0.001). Moreover, in the animals from the experimental groups (HR*_Amph_* and LR*_Amph_*) compared to the animals from the control groups (HR*_ACSF_* and LR*_ACSF_*), the differentiation of cFos protein was also observed in the remaining structures of the thalamus belonging to the nonspecific arousal system. These were the following nuclei: the mediolateral (CL) (LR group: z = −2.366; *p* < 0.05), centralo-medial (CM) (LR group: z = −2.746; *p* < 0.01), inter anteromedial (IAM) (LR group: z = −1.960; *p* < 0.05), paracentral (PC) (HR group: z = −2.521; *p* < 0.05) (LR group: z = −2.666; *p* < 0.01), paraventricular (PVA) (LR group: z = −2.118; *p* < 0.05), reuniens (Re) (HR group: z = −2.756; *p* < 0.05) and reticular nuclei (Rt) (HR group: z = −2.845; *p* < 0.01).

**Table 5:** Density of cFos+ cell nuclei (number/1 mm^2^) (mean ±SE) in the thalamus nuclei: anterodorsal (AD), anteromedial (AM), anteroventral (AV) and mediodorsal (MD) nuclei, lateral (LHb) and medial (MHb) habenula, mediolateral (CL), medial (CM), inter anteromedial (IAM), inter mediodorsal (IMD), paracentral (PC), paratential (PT) and paraventricular (PVA) nuclei, reuniens (Re), rhomboid (Rh) and reticular thalamus nuclei (Rt) in the brains of rats after novelty test from the experimental (HR*_Amph_*; n = 5; and LR*_Amph_*; n = 5) and control (HR*_ACSF_*; n = 5; and LR*_ACSF_*; n = 5) groups.

Thalamus	Control Groups		Experimental Groups	
	HR*_ACSF_*	LR*_ACSF_*	HR*_Amph_*	LR*_Amph_*
AD	53.39 ± 8.80	32.03 ± 3.26	117.55 ± 12.27 ^*,γ,&&^	78.73 ± 6.96 ^##^
AM	23.79 ± 5.78	10.71 ± 2.98	64.12 ± 8.46 ^*,&^	45.39 ± 10.95 ^#^
AV	12.51 ± 1.81	7.28 ± 0.91 ^*^	116.90 ± 11.03 ^***,γγγ,&&&^	33.65 ± 3.56 ^###,$$$^
MD	27.08 ± 6.40	23.61 ± 4.93	118.66 ± 19.06 ^**,γ,&&&^	45.87 ± 6.84 ^#^
LHb	72.16 ± 11.65 ^††^	11.70 ± 3.25	104.11 ± 21.62	228.01 ± 37.16 ^#,$^
MHb	72.18 ± 8.75 ^††^	24.03 ± 4.25	403.33 ± 66.37 ^*,&^	242.26 ± 24.30 ^###,$$$^
CL	22.38 ± 9.12	16.08 ± 2.59	98.34 ± 25.19	107.50 ± 13.14 ^#,$^
CM	48.61 ± 16.12	14.85 ± 3.08	87.00 ± 52.44 ^&^	41.15 ± 5.90 ^##^
IAM	26.78 ± 6.68 ^†^	7.30 ± 1.46	22.21 ± 4.16 ^&&^	13.78 ± 2.87 ^#^
IMD	57.48 ± 9.36 ^††^	11.18 ± 4.50	89.37 ± 34.98	27.33 ± 6.34
PC	21.18 ± 5.32	10.81 ± 1.97	103.37 ± 21.05 ^*,γ,&^	53.76 ± 6.99 ^##,$^
PT	24.61 ± 10.79 ^†^	7.23 ± 1.62	88.81 ± 38.16 ^γ,&&^	20.18 ± 3.58
PVA	170.27 ± 34.00 ^†,$$^	47.59 ± 8.55	96.49 ± 26.84 ^&&^	87.35 ± 19.12 ^#^
Re	14.81 ± 2.91	14.58 ± 2.85	100.13 ± 36.73 ^*,&&^	16.95 ± 3.39
Rh	47.22 ± 13.06 ^†^	14.09 ± 3.47	114.78 ± 49.36 ^&^	37.02 ± 7.38
Rt	31.70 ± 7.38 ^††^	16.90 ± 4.35	103.82 ± 15.23 ^**,γγ,&&^	27.18 ± 5.18

Explanations: *** *p* < 0.001, ** *p* < 0.01 and * *p* < 0.05, differences between the HR*_Amph_* and HR*_ACSF_*; ### *p* < 0.001, ## *p* < 0.01 and # *p* < 0.05, differences between the LR*_Amph_* and LR*_ACSF_*; γγγ *p* < 0.001, γγ *p* < 0.01 and γ *p* < 0.05, differences between the HR*_Amph_* and LR*_Amph_*; †† *p* < 0.01 and † *p* < 0.05, differences between the HR*_ACSF_* and LR*_ACSF_*; &&& *p* < 0.001, && *p* < 0.01 and & *p* < 0.05, differences between the HR*_Amph_* and LR*_ACSF_*; $$$ *p* < 0.001, $$ *p* < 0.01 and $ *p* < 0.05, differences between the HR*_ACSF_* and LR*_Amph_* (Wilcoxon’s signed rank test).

Table 6 presents the results concerning the density of cFos+ protein in selected sub- and hypothalamic structures. The highest density of cFos in the HR group was observed in the LH and the zona incerta (ZI) (*p* < 0.01) (HR*_Amph_* vs. HR*_ACSF_*: LH: z = −3.154; ZI; z = −2.666) and in the anterior hypothalamus (AH) and paraventricular nucleus (PVN) (*p* < 0.05) (HR*_Amph_* vs. HR*_ACSF_*: AH: z = −2.521; PVN: z = −2.380). In LR animals (LR*_Amph_* vs. LR*_ACSF_*), the highest cFos protein density was observed in the LH (*p* < 0.001) (z = −3.408) and to a lesser extent in the AH and ZI (*p* < 0.01) (AH: z = −2.746; ZI: z = −3.110), and in the posterior hypothalamus (PH), dorsal part of the dorsomedial nucleus (DMD), and in the supraoptic (SO) and arcuate nuclei (Arc) (*p* < 0.05) (PH: z = −2.023; DMD: z = −2.366; SO: z = −2.100 and Arc: z = −2.310).

**Table 6:** Density of cFos+ cell nuclei (number/1 mm^2^) (mean ±SE) in the hypothalamus nuclei: anterior (AH), dorsal (DA) and paraventricular (PVN) nuclei, lateral (LH) and posterior (PH) hypothalamus, dorsomedial (DMD), ventromedial (VMH), supraoptic (SO) and arcuate (Arc) nuclei and zona incerta (ZI) in the brains of rats after novelty test from the experimental (HR*_Amph_*; n = 5; and LR*_Amph_*; n = 5) and control (HR*_ACSF_*; n = 5; and LR*_ACSF_*; n = 5) groups.

Hypothalamus and Subthalamus	Control Groups		Experimental Groups	
	HR*_ACSF_*	LR*_ACSF_*	HR*_Amph_*	LR*_Amph_*
AH	29.48 ± 6.96 ^†^	10.64 ± 1.92	131.33 ± 34.58 ^*,γγ,&&^	35.73 ± 4.17 ^##^
DA	45.58 ± 3.71	16.44 ± 3.27	100.85 ± 26.31	108.58 ± 5.10
PVN	111.59 ± 28.53	94.57 ± 18.14	270.87 ± 49.56 ^*,γ,&^	105.91 ± 11.47
LH	25.19 ± 5.81 ^††^	12.22 ± 2.88	88.35 ± 11.81 ^**,&&&^	67.00 ± 11.60 ^###,$$^
PH	33.43 ± 10.70 ^††^	10.42 ± 2.18	157.11 ± 65.38	74.49 ± 10.93 ^#,$^
DMD	70.55 ± 12.75 ^†^	13.66 ± 3.25	198.45 ± 11.34	121.89 ± 13.75 ^#,$^
VMH	74.84 ± 12.83 ^††^	24.51 ± 11.72	165.16 ± 57.02 ^&&^	121.31 ± 42.22
SO	335.61 ± 61.49	232.66 ± 31.99	447.19 ± 33.95 ^&^	458.28 ± 50.92 ^#^
Arc	121.52 ± 22.22 ^†^	60.54 ± 15.03	371.88 ± 99.11 ^&&^	196.81 ± 30.94 ^#^
Zi	18.23 ± 4.39	10.88 ± 2.00	97.54 ± 13.99 ^**,&&^	104.44 ± 19.03 ^##,$$^

Explanations: ** *p* < 0.01 and * *p* < 0.05, differences between the HR*_Amph_* and HR*_ACSF_*; ### *p* < 0.001, ## *p* < 0.01 and # *p* < 0.05, differences between the LR*_Amph_* and LR*_ACSF_*; γγ *p* < 0.01 and γ *p* < 0.05, differences between the HR*_Amph_* and LR*_Amph_*; †† *p* < 0.01 and † *p* < 0.05, differences between the HR*_ACSF_* and LR*_ACSF_*; &&& *p* < 0.001, && *p* < 0.01 and & *p* < 0.05, differences between the HR*_Amph_* and LR*_ACSF_*; $$ *p* < 0.01 and $ *p* < 0.05, differences between the HR*_ACSF_* and LR*_Amph_* (Wilcoxon’s signed rank test).

In the VTA in HR animals (HR*_Amph_* vs. HR*_ACSF_*), more cFos was observed in the PBP (z = −3.574; *p* < 0.001), IF (z = −2.240; *p* < 0.05) and Rli (z = −2.201; *p* < 0.05). In LR animals (LR*_Amph_* vs. LR*_ACSF_*), this differentiation included only the periaqueductal gray (PAG) (z = −2.023; *p* < 0.05) (Table 7).

**Table 7:** Density of cFos+ cell nuclei (number/1 mm^2^) (mean ±SE) in the limbic structures of the midbrain: parabrachial pigmentosus (PBP), paranigral (PN) and interfascicularis (IF) nuclei, raphe linearis pars dorsalis (Rli) and periaqueductal gray (PAG) in the brains of rats after novelty test from the experimental (HR*_Amph_*; n = 5; and LR*_Amph_*; n = 5) and control (HR*_ACSF_*; n = 5; and LR*_ACSF_*; n = 5) groups.

Limbic Structures of the Midbrain	Control Groups		Experimental Groups	
	HR*_ACSF_*	LR*_ACSF_*	HR*_Amph_*	LR*_Amph_*
PBP	17.96 ± 4.69	18.28 ± 3.51	116.42 ± 36.76 ^***,&&&^	53.96 ± 14.92
PN	36.37 ± 7.82	16.67 ± 2.89	56.40 ± 8.33 ^&&^	185.19 ± 46.91
IF	36.14 ± 10.01	17.80 ± 4.01	91.83 ± 15.50 ^*,&^	56.61 ± 18.85
Rli	9.07 ± 2.32	7.72 ± 1.25	335.94 ± 102.04 ^*,&^	30.15 ± 12.65
PAG	20.99 ± 4.73	9.39 ± 1.34	70.23 ± 7.73 ^&^	97.26 ± 32.87 ^#^

Explanations: *** *p* < 0.001 and * *p* < 0.05, differences between the HR*_Amph_* and HR*_ACSF_*; # *p* < 0.05, differences between the LR*_Amph_* and LR*_ACSF_*; &&& *p* < 0.001, && *p* < 0.01 and & *p* < 0.05, differences between the HR*_Amph_* and LR*_ACSF_* (Wilcoxon’s signed rank test).

Table 8 presents the remaining structures in the extrapyramidal and ventral striatum. Also here, a higher density of cFos+ protein was found in all analyzed structures in HR animals (HR*_Amph_* vs. HR*_ACSF_*): the Acb (z = −3.296; *p* < 0.001), caudate putamen (Cpu) (z = −4.163; *p* < 0.001), globus (GP) (z = −2.366; *p* < 0.05) and VP (z = −2.073; *p* < 0.05), and in the SNC (z = −4.286; *p* < 0.001) and SNR (z = −3.180; *p* < 0.001), as well as in LR (LR*_Amph_* vs. LR*_ACSF_*): the Acb (z = −3.621; *p* < 0.001), Cpu (z = −5.310; *p* < 0.001), GP (z = −2.100; *p* < 0.05), VP (z = −3.010; *p* < 0.01) and the SNC and SNR (z = −2.023; *p* < 0.05).

In addition, Table 1, Table 2, Table 3, Table 4, Table 5, Table 6, Table 7 and Table 8 additionally present the differences between the experimental group (HR*_Amph_* vs. LR*_Amph_*) and the control group (HR*_ACSF_* vs. LR*_ACSF_*) and the comparisons between the experimental and control groups (HR*_Amph_* vs. LR*_ACSF_* and HR*_ACSF_* vs. LR*_Amph_*) (Wilcoxon’s signed rank test).

**Table 8:** Density of cFos+ cell nuclei (number/1 mm^2^) (mean ±SE) in the extrapyramidal and ventral striatum: nucleus accumbens (Acb), caudate putamen (Cpu), globus (GP) and ventral (VP) pallidum and substantia nigra, pars compacta (SNC) and reticulata (SNR) in the brains of rats after novelty test from the experimental (HR*_Amph_*; n = 5; and LR*_Amph_*; n = 5) and control (HR*_ACSF_*; n = 5; and LR*_ACSF_*; n = 5) groups.

Extrapiramidal Structures and Ventral Striatum	Control Groups		Experimental Groups	
	HR*_ACSF_*	LR*_ACSF_*	HR*_Amph_*	LR*_Amph_*
Acb	19.75 ± 9.70	6.16 ± 0.75	85.23 ± 14.64 ^***,&&&^	58.11 ± 4.26 ^###,$$^
Cpu	28.18 ± 5.58 ^†^	5.84 ± 0.85	88.85 ± 9.74 ^***,&&&^	59.08 ± 12.45 ^###^
GP	19.07 ± 2.52 ^†^	5.71 ± 1.03	55.12 ± 12.65 ^*,γ,&^	16.21 ± 2.51 ^#^
VP	37.12 ± 6.00 ^†††^	11.99 ± 1.74	82.55 ± 17.62 ^*,γ,&^	40.78 ± 5.94 ^##^
SNC	24.02 ± 5.82	24.13 ± 4.36	236.64 ± 24.00 ^***,γ,&&&^	96.35 ± 11.21 ^#^
SNR	11.07 ± 2.09	12.13 ± 2.19	131.92 ± 17.22 ^***,&&&^	46.89 ± 13.96 ^#^

Explanations: *** *p* < 0.001 and * *p* < 0.05, differences between the HR*_Amph_* and HR*_ACSF_*; ### *p* < 0.001, ## *p* < 0.01 and # *p* < 0.05, differences between the LR*_Amph_* and LR*_ACSF_*; γ *p* < 0.05, differences between the HR*_Amph_* and LR*_Amph_*; ††† *p* < 0.001 and † *p* < 0.05, differences between the HR*_ACSF_* and LR*_ACSF_*; &&& *p* < 0.001 and & *p* < 0.05, differences between the HR*_Amph_* and LR*_ACSF_*; $$ *p* < 0.01, differences between the HR*_ACSF_* and LR*_Amph_* (Wilcoxon’s signed rank test).

### 2.3. Histological Analysis

**Figure 10:**
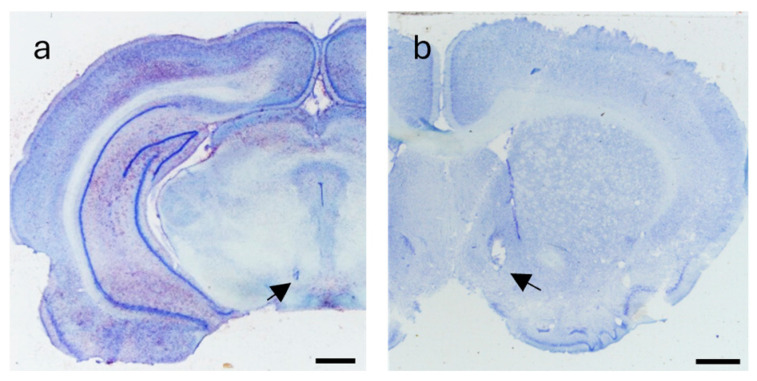
Photographs of select brain sections from representative rats in the experimental group (HR*_Amph_*) stained with Nissl staining with localization of the electrode in the ventral tegmental area (VTA) (distance from bregma to posterior: −5.04 mm; black arrow—the position of the electrode tip) (**a**) and the cannula in the nucleus accumbens shell (AcbSh) (distance from bregma to anterior: +2.04 mm; black arrow—the position of the cannula tip) (**b**) according to the rat brain atlas [42] (a magnifier Stemi 508; Zeiss with camera Axiocam 105 color; Zeiss and with integrated software Zen Digital Imaging (version 3.2 Blue Edition); Zeiss, magnification 2.5 × 10, scale bar 1 mm—right lower corner of the photos).

All animals tested in the experimental (n = 10) and control (n = 10) groups had the proper position of the stimulation electrodes in the VTA and the appropriate position of the injection cannula tips in the AcbSh. The VTA stimulation tips were localized between 4.80 and 5.80 mm caudally from the bregma (Figure 10a), and cannula tips were localized between 2.28 and 1.08 mm rostrally from the bregma (Figure 10b) according to rat atlas [42].

## 3. Discussion

In the following study, using a model of induced feeding by electrical stimulation of the VTA, we provide evidence concerning the finding that rats with higher behavioral activity (responding more strongly to novelty) and increased stress sensitivity show faster feeding responses (shortened iFR latencies) and different neuronal excitation (higher TH+ and diverse ChAT+ cell density activity and higher detection of cFos+ neurons) in selected cortical and subcortical structures to addictive agents (single amphetamine injection into the AcbSh). The injection in the AcbSh was performed unilaterally, ipsilateral to the stimulated hemisphere in the VTA.

Our previous studies have described the behavioral responses assessed using Es-VTA (increase in locomotor activity, exploration, food intake, or other) in detail [43,44,45]. Food intake, accompanied by general arousal, is the most prominent effect of VTA activation [43,46,47]. Only rats that demonstrated a reliable response to food were included in this experiment. The effect of amphetamine injection into the AcbSh on iFR was analyzed based on feeding latencies and left or right shifts of the latency/frequency curve. The iFR model used here allowed us to efficiently and reliably determine the adaptive capacity of the test animals (facilitation as a shift to the left seen in HR*_Amph_* rats or impairment as a shift to the right in LR*_Amph_* rats of the observed behavioral response) to the new stimulus.

The VTA, which primarily projects to the Acb, represents a dopamine-dependent neuronal circuit mediating reward and motivational aspects of both natural and pathological behavior (e.g., [48,49,50]). Among other things, AcbSh is involved in emotional functions and feeding control [51,52]. In our study, we found differential variation in motivational behavior correlated with locomotor activity score in the novelty test after amphetamine injection into the AcbSh. In HR*_Amph_* animals, a shortening of the iFR latency was observed, whereas in LR*_Amph_* animals, it was prolonged. This effect was also observed relative to the control, which was the injection of ACSF (HR*_ACSF_* and LR*_ACSF_*) into the AscbSh.

In the current study, we also analyzed the density of the most critical dopaminergic cell groups of the hypothalamus (A15–A11) and the midbrain (A10–A8), cholinergic (Ch1–Ch6), and cFos protein in selected cortical and subcortical structures. In previous studies, we found that there are differences in the topography of active dopaminergic cells in the midbrain in A9–A10 [39], A8 [40], and the hypothalamus [41] in HR and LR rats. In these animals, we may relate to different behaviors in a new environment and reactions to psychoactive substances [21]. Moreover, it is known that these animals also differ in the level of dopamine release in the Acb (higher in HR) and the PFC (higher in LR) [20,30,31,53,54]. In the present study, we decided not only to activate the mesolimbic system by electrical stimulation of the VTA but also to additionally enhance this response by injection of amphetamine directly into the AcbSh nucleus. We found that in the rats from the experimental group (amphetamine injection), there was a significant increase in the density of TH+ cells compared to the control group (ACSF injection), which was analyzed together in all groups A15–A8. Moreover, a significant increase in the density of TH+ cells was found in the HR*_Amph_* animals compared to the LR*_Amph_* animals. Analysis of individual dopaminergic groups confirmed the differences in the density of TH+ cells, especially in groups A12–A11 in the hypothalamus and A10 and A8 in the midbrain. The exception was the A10 group in the SN, particularly in SNC and SNR, where a higher density of TH+ cells was observed in the control group. This diversity of activation of dopaminergic cells in the hypothalamus and midbrain in animals differing in the level of spontaneous locomotor activity may suggest that the same stress stimulus/drug activates different neuronal projections of the hypothalamus and midbrain, specific for each of the two types of animal behavior [55]. AcbSh controls ingestive behavior through direct or indirect neural interconnections, primarily with LH [56]. LH is a structure that plays a crucial role in the regulation of feeding. It is the convergence point of many peripheral signals and neural pathways from the brainstem and higher cortical centers controlling energy homeostasis and body weight [57]. Moreover, stimulation of this structure also elicits responses to both food and reward [58]. In addition, there are also several other important regions in the hypothalamus related to food intakes, such as the Arc (A12-type dopaminergic cells), the DMD (A13-type dopaminergic cells), the PVN, and the ventromedial hypothalamus (VMH) (A14-type dopaminergic cells), which together with the LH, through the production of orexigenic or anorexigenic neurotransmitters or neuromodulators [57,59,60,61], participate in the regulation of appetite and energy balance [62]. An example is ghrelin, produced in the stomach’s endocrine cells [63] and synthesized near the third ventricle between areas involved in feeding and energy metabolism [57]. Direct injection of this compound into the cerebral ventricles increased *c-fos* expression in the Arc, PVN, DMD, and LH. Insulin administration also significantly increased the expression of this protein in these hypothalamic nuclei [57]. However, these studies did not account for individual differences in animals’ responses to novelty. Histochemical studies found that in group A12, located within the Arc, more TH mRNA was found in rats with higher locomotor activity compared to rats with lower locomotor activity [64]. Also, our previous immunohistochemical studies found a correlation in the hypothalamus between the total number of TH+ cells and the locomotor activity score in the novelty test. They were found for the entire A11–A15 region and separately for A11, A12, and A14, but not for dopaminergic A13 and A15 cells [41]. In the present study, after VTA stimulation and amphetamine injection into the AcbSh, the most significant differentiation within TH+ was observed only in A11 cells and, to a lesser extent, in A12 in HR and LR animals. Analysis of cFos confirmed its increased density in the hypothalamus, especially in AH, PVN, PH, DMD, Arc, Zi, and LH.

It is known that the largest concentration of dopaminergic neurons is in the midbrain, and they are located mainly in three nuclei: VTA (A10), RRF (A8), and SNC (A9), as well as sparsely scattered in the SNR. The two main subnuclei of the VTA are the PBP and PN nuclei. In addition, the midline nuclei IF, Rli, and caudal nucleus (Cli) are often considered subregions of the VTA [65,66,67,68,69]. In turn, the RRF is located dorsal and caudal to the SN and has both dopaminergic [70,71,72] and nondopaminergic [66] neurons and projects mainly to the dorsal and ventral striatum, piriform and entorhinal cortex [73], and amygdala [74]. These three main structures—the VTA, SN, and RRF—are involved in three complex circuits: the mesostriatal, mesolimbic, and mesocortical pathways. These pathways involve behavioral manifestations, motor skills, learning, reward, and neurodegenerative diseases [75]. Our studies showed that in HR*_Amph_* animals, the highest TH+ density was primarily in the midbrain in the VTA, specifically in the PN and PBP. According to Lammel et al. [76], VTA dopaminergic neurons located in the medial posterior part of the PN and PBP selectively project to the medial part of the AcbC and AcbSh, the medial prefrontal cortex (mPFC), and the BLA, whereas VTA dopaminergic neurons located in the lateral posterior and anterior part of the PBP mainly project to the lateral part of the AcbSh [76,77,78,79,80]. In our experiments, we targeted Es-VTA into the lateral anterior part of the VTA in the PBP and the amphetamine injection to the lateral part of the AcbSh. Moreover, cFos analysis also revealed a significant increase in its density primarily in limbic structures of the cortex (CG1, CG2, RSG, and RSA), Acb, and thalamus (AD, AM, AV, and MD) in HR*_Amph_* and LR*_Amph_* animals, and in the amygdala (ACo, BLA, BST, Ce, and Me) with a predominance of differentiation in LR*_Amph_* animals.

An interesting observation is the intermediate-level increase in TH+ density in the RRF compared to SN. This finding suggests an indirect role for the RRF in transmitting excitation between the VTA and the SN. Another interesting result is the level of TH+ density in the SN, where the opposite effect (decrease in the level of TH+ density) was observed in HR and LR animals after amphetamine injection compared to the control. This effect was evident in the SNC and SNR. Moreover, the results concerning the detection of cFos protein showed a significant increase in the SNC and SNR, primarily in the HR*_Amph_* animals. Recent literature suggests that dopaminergic neurons in the SNC, as in the VTA, exhibit functional heterogeneity [81,82,83] correlated with differences in dendritic architecture and afferent connectivity [82], which may contribute to their diverse behavioral roles [76]. Furthermore, SNC dopaminergic cells also release GABA, inhibiting the striatum’s dorsal medium spike neurons [84]. Because this GABA release depends on the vesicular monoamine transporter, vMAT2, other subpopulations of dopaminergic neurons may also co-release GABA [76]. In previous studies using the same iFR assessment by using the Es-VTA method together with the injection of an antagonist (MK-801) and an agonist (NMDA) of glutamatergic receptors into the PPN nucleus, we also obtained a significantly smaller number of TH+ in both the SN and the VTA and no cFos activation within these structures compared to the control [44]. In turn, stimulation (morphine) or inhibition (naloxone) of opioid receptors in the PPN caused an increase in the detection of cFos protein in the SN, primarily in the SNR and to a lesser extent in the SNC, and in the VTA, mainly in the PBP and Rli [45]. However, these studies did not consider the differentiation of animals in terms of behavioral activity. Thus, both the current (amphetamine injection into AcbSh and Es-VTA) and earlier (morphine or naloxone injection into PPN and Es-VTA) behavioral and neuronal findings suggest the activation or blockade of distinct neuronal projections and the activation of various subpopulations of dopaminergic neurons in the VTA, which may result in different behavioral responses to addiction in animals exhibiting different sensitivities to different stressful stimuli.

In the current study, a single injection of amphetamine directly into the AcbSh was used because, according to literature data [14], after chronic pharmacological stimulation of the mesolimbic system, such as amphetamine self-administration, no differences in TH mRNA levels were found in HR and LR animals in both the SN and VTA. However, a significant increase in DAT mRNA levels was observed in the VTA and SN in HR rats but not in LR rats compared to controls. It is known that the regulation of DAT is directly related to the regulation of dopamine in the synaptic clefts. DAT is located on the presynaptic axonal terminals and projection bodies of VTA dopaminergic neurons to the Acb, responsible for regulating extracellular dopamine levels via cytoplasmic reuptake of neurotransmitters [85]. Amphetamine-binding DAT prevents dopamine reuptake and promotes the reversal of dopamine transport into the extracellular environment, increasing available free catecholamine [86]. Thus, the increase in DAT levels in dopaminergic neurons projecting to the Acb may be a consequence of amphetamine [14,87], which may explain in our study the shortened iFR latency in HR*_Amph_* animals and the increase in TH+ in response to amphetamine injection into the AcbSh. Furthermore, the increase in DAT mRNA levels in HR rats, but not in LR rats, may contribute to the greater addiction susceptibility of HR rats [14]. However, the molecular mechanism of regulation of DAT gene expression during chronic amphetamine administration is still not fully understood [14] because the increase in DAT mRNA levels is not necessarily associated with an increase in the dopamine decarboxylase enzyme (AADC) itself, as well as the vMAT2 and dopamine itself [67]. However, after chemical damage in the striatum and olfactory deprivation in the olfactory colliculus in transgenic mice, a rapid increase in TH+ cells is detected by immunohistochemical methods [67,88]. Moreover, it is known that molecular relationships, e.g., high DAT density combined with amphetamine self-administration, cause a rapid induction of the intracellular response for early cellular response genes. This involves the induction of specific transcription factors for *c-fos* and CREB, such as cyclic adenosine monophosphate (cAMP), cyclic dependent kinase (CDK5), and tyrosine kinase [89,90,91].

Chronic amphetamine abuse is known to damage the hippocampus, which is a brain region involved in learning and memory [92] and associated with addiction development [93]. In animal studies, a detailed analysis of the hippocampal structure at the molecular level was performed after the novelty test within the moss fibers (SP-MF) of the CA3 pyramidal cell, where HR individuals showed significantly less SP-MF in the upper parts of the single dendritic axis of this cell [94]. Furthermore, HR animals at rest showed a lower affinity of steroids for glucocorticoid receptors (GR) in the CA1 field [23], a different activation of serotonergic 5-HT1A receptors in the hippocampus [95], and also phenotypic differences in the mRNA signal for 5-HT6 were found, where in LR rats, they were statistically higher in the most anterior region of the dentate gyrus and HR rats in the middle areas of the dentate gyrus [96]. In HR rats, 5-HT7 transcript levels were significantly lower than those in LR rats within the dorsal hippocampus and regions associated with the thalamocortical projection [96]. Therefore, there may be a different route of propagation and processing of impulses in the hippocampus under the influence of stressful stimuli in these animals [94]. Long-term amphetamine administration in rats significantly increased cFos expression in the Acb and VTA. At the same time, no significant changes were observed in other regions such as the cerebral cortex, Cpu, hippocampus, amygdala, or habenula [97]. However, the above studies considered only one factor: the animals’ variation to novelty or addiction to psychostimulants. In the present study, were taken into account both the individual variability of animals to stress and the activity of the ML by unilateral Es-VTA and unilateral injection of amphetamine into the Acb. It was found that unilateral injection of amphetamine into the Acb together with Es-VTA did not cause differences in cFos density in the hippocampus, both in the DG and the CA1–CA3 fields, in HR and LR animals compared to controls. Perhaps the lack of neuronal activation in the hippocampus may be due to the action of other significant projections, e.g., serotonergic, glutamatergic, or cholinergic.

In the present study, we also analyzed the cell density of the most critical cholinergic groups after amphetamine injection into the AcbSh and Es-VTA. The cholinergic nuclei of the central nervous system are grouped in the forebrain (Ch1–Ch4) and brainstem (Ch5–Ch6) [98]. From the septal nuclei (Ch1–Ch3), cholinergic projections go primarily to the hippocampus, where they meet dopaminergic (from the VTA), serotonergic (from the raphe nucleus), and noradrenergic (from the locus coeruleus) pathways [98]. The basal nucleus of Meynert in the forebrain is the major cholinergic nucleus in the brain (Ch4), from which cortical projections primarily project [99]. Cholinergic projections originating in the brainstem (Ch5–Ch6) project to multiple areas, including the prefrontal cortex, basal forebrain, thalamus, hypothalamus, amygdala, and hippocampus [100,101,102], and the pathway to the VTA modulates dopamine release in the VTA [103], thus participating in the mechanism responsible for the development of addiction. Cholinergic neurons in the central nervous system modulate attention [104,105], learning [106,107,108], memory [109,110], and motivation [111]. Cholinergic cells in the forebrain are located mainly in the subcortical nuclei [112] and are characterized by local and distal communication networks [113]. This is confirmed by studies in which cholinergic denervation of the neocortex in the rat led to a dramatic enhancement in dopamine release in the Acb, but cholinergic damage in the striatum did not increase its release, which was additionally enhanced by amphetamine injection [114]. An increased rate of acetylcholine turnover in the hippocampus was found after chemical damage with 6-hydroxydopamine of A10 dopaminergic cells or their endings in the septum [115]. Thus, dopaminergic neurons exert a tonic inhibitory effect on cholinergic metabolism in the septohippocampal pathway. In the present study, the analysis of the density of the main cholinergic groups Ch1–Ch6 together showed a difference in the density of ChAT+ cells and the locomotor activity of the studied animals in both the control group (HR*_ACSF_* vs. LR*_ACSF_*) and the experimental group (HR*_Amph_* vs. LR*_Amph_*). However, amphetamine injection alone did not cause a significant difference in ChAT+ density in animals with the same locomotor activity (HR*_Amph_* vs. HR*_ACSF_* and LR*_Amph_* vs. LR*_ACSF_*). Analysis of individual Ch1–Ch6 groups showed a higher ChAT+ density after amphetamine injection in the AcbSh only in groups Ch5 in LR animals and Ch6 in HR animals. Therefore, it can be assumed that in animals more reactive to a stressful stimulus (e.g., novelty), changes in dopaminergic projections to the forebrain influence changes in its cholinergic projections, which in turn can only induce regionally selective changes in the control of dopaminergic systems, in contrast to cholinergic projections from the brainstem. However, it seems that these regional changes in dopaminergic projections can significantly influence the significant increase in dopamine release induced by VTA stimulation and additionally enhanced by amphetamine injection in the Acb. In addition, cholinergic activity changes can lead to glutamatergic transmission. Dopaminergic neurons are controlled by glutamatergic neurons of the prefrontal cortex either directly or indirectly [116,117], acting as accelerators or brakes, respectively [114]. If dopamine release is disturbed, e.g., by amphetamine, a negative feedback loop is activated, leading to a much more significant effect on the “brake,” thereby reducing dopamine release [118]. However, it is not fully understood how much of the “brake” is activated in individuals with increased or decreased stress sensitivity.

In summary, we conclude that in animals with different locomotor activity, not only is variation observed in activated mesolimbic dopaminergic function, but this variation is also observed in the cholinergic system, significantly when the reward system is activated not only by electrical stimulation alone but additionally enhanced by amphetamine injection directly into the AcbSh. Furthermore, our results show that the mechanisms of neuronal activity of the VTA, its projections, and relationships between other brain structures may be correlated with individual behavioral and neurochemical differences, which are yet not fully understood.

## 4. Materials and Methods

**Figure 11:**
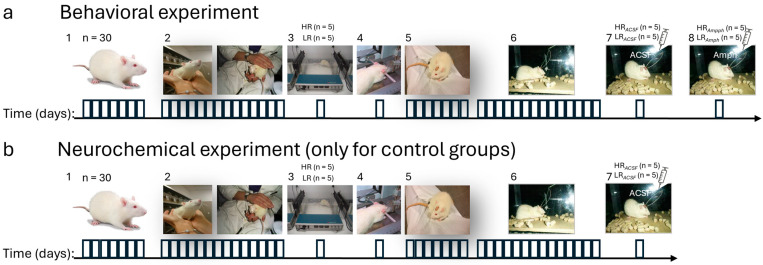
Schematic diagrams of all experimental behavioral procedures conducted on rats (n = 60) during behavioral (n = 30) (**a**) and neurochemical experiments (n = 30) (**b**) together with the division of animals into control (n = 10) and experimental groups (n = 10). Explanations: (1) acclimatization of animals, (2) handling, this procedure continued throughout the cycle of behavioral procedures, (3) novelty test, (4) stereotaxic surgery of animals for implantation of a stimulating electrode into the ventral tegmental area (VTA) and an injection cannula into the nucleus accumbens shell (AcbSh), (5) recovery period with handling procedure, (6) screening electrical stimulation of the VTA (Es-VTA) to individually select current parameters for each animal and determine excitability threshold (analysis of the latency of the induced food response (iFR)), (7) injection of artificial cerebrospinal fluid (ACSF) (0.5 µL) into the AcbSh—the control groups of rats (HR*_ACSF_*; n = 5 and LR*_ACSF_*; n = 5) in behavioral (a) and neurochemical experiments (b; only the brains of these rats were used as control groups (HR*_ACSF_*; n = 5 and LR*_ACSF_*; n = 5) for these studies), (8) injection of amphetamine (5.0 µg/µL) into the AcbSh—the experimental groups of rats (HR*_Amph_*; n = 5 and LR*_Amph_*; n = 5) in behavioral (**a**). The brains of rats from the behavioral studies (**a**) were used as experimental groups (HR*_Amph_*; n = 5 and LR*_Amph_*; n = 5) for neurochemical studies (**b**).

All animal experiments were carried out by the European Communities Council Directive of 24 November 1986 (86/609/EEC) and under the authority of the Local Ethical Committee (No 15/2014). All efforts were made to minimize the discomfort and the number of animals. Studies were performed using male Wistar rats (Tri-City Central Animal Laboratory, Research and Service Centre of the Medical University of Gdansk, Poland, n = 60) weighing approximately 200 ± 60 g at arrival. During the experiment, rats were housed separately in polycarbonate cages (20 cm width, 37 cm length, 18 cm height) on a 12 h light/dark cycle (lights on at 06:00) in an air-conditioned, constant-temperature (22 ± 2 °C) room could visually observe other subjects and were indirectly exposed to different subjects’ cage odors. Water and food (standard compound feed for laboratory animals, Labofeed, Poland) were available ad libitum. Upon arrival at the animal quarters, animals were allowed to adapt to the laboratory conditions for one week before the beginning of the experimental procedure. Then, the handling procedure was carried out daily for two weeks for about 5 min for each animal, as previously described [39,41]. For each group of rats in the experimental and control groups, the behavioral experimental procedures described below are summarized in Figure 11.

### 4.1. Novelty Test

The level of spontaneous locomotor activity in response to novelty was determined in accordance with the method of [19], with some modifications later described [21,39,41]. The novel environment was a clear Plexiglas cage (430 mm × 430 mm × 200 mm) equipped with 15 photoelectric cells placed on both axes of the cage (an actinometer Opto Varimex Minor—Columbus, OH, USA). The cage automatically recorded rats’ locomotor activity (horizontal, vertical, and ambulatory plane photocell counts) (Figure 11(a3,b3)). The rats (n = 60) were placed individually in the actometer for 2 h (4.00–6.00 p.m.). Only the rats with upper (HR; n = 10) and lower index (LR; n = 10) for the novelty test were used for further studies. The rats from each locomotor activity group were randomly assigned to behavioral and neurochemical studies (Figure 11). For the behavioral study (n = 10), just before Es-VTA, HR (n = 5) and LR (n = 5) rats received ACSF injections into the AcbSh as a control (HR*_ACSF_* and LR*_ACSF_* as *Acsf* groups), followed by amphetamine injections into the AcbSh the next day as an experiment (HR*_Amph_* and LR*_Amph_* as *Amph* groups). This study analyzed and compared only the behavior of the *Acsf* and *Amph* groups (Figure 11(a1–a8)). Subsequently, the brains of these rats were used for neurochemical analyses and were the experimental groups here (HR*_Amph_*; n = 5 and LR*_Amph_*; n = 5). Additionally, for neurochemical studies, a control group was established (n = 10). For this purpose, rats were subjected to the same behavioral procedures using the Es-VTA as in the animals used for the behavioral studies, but only with an injection of ACSF into the AcbSh (HR*_ACSF_*; n = 5 and LR*_ACSF_*; n = 5). This group was used exclusively to collect brain samples for immunofluorescence staining as a control group (Figure 11(a1–a7)).

The remaining rats with intermediate locomotor activity (n = 40) were not used for further experiments.

### 4.2. Surgery

The surgery (Figure 11(a4,b4)) used 1–2.5% isoflurane (Aeranne; Baxter, Deerfield, IL, USA) anesthesia. All rats involved in behavioral and neurochemical procedures (n = 20) underwent unilateral implantation of a stimulation electrode in the VTA, unilateral placement of an injection cannula in the AcbSh nucleus, and placement of a reference electrode on the skull surface. Referenced and stimulation electrode preparation methods were described previously [43,45]. The coordinates for implantation of stimulation electrodes for VTA were 4.80–5.20 mm posterior to the bregma, 1.00 mm lateral to the midline, and 8.00–8.10 mm ventral to the skull surface (skull leveled) [45]. The coordinates for implantation of injection cannulas for the AcbSh were 1.00–2.16 mm anterior to the bregma, 0.90–1.20 mm lateral to the midline, and 7.00–7.10 mm ventral to the skull surface (skull leveled) [47]. After surgery, all rats underwent a 7-day recuperation period (Figure 11(a5,b5)). Subsequent behavioral procedures involving electrical stimulation of the VTA and drug injections into the AcbSh nucleus were only conducted after the rats had fully recovered.

### 4.3. Behavioral Procedures

Subsequent behavioral procedures involving electrical stimulation of the VTA (Es-VTA), assessment of the latency of the induced food reaction (iFR), and injection of drugs (ACSF/Amph) into the AcbSh nucleus were performed only after recuperation (7 days after surgery) in all rats (n = 20) (Figure 11(a5,b5)). Previous studies described these procedures in detail [43,44,45].

As described in the “*Novelty test*” subsection, before the stimulation procedure, all animals (n = 10) from the behavioral studies (Figure 11(a7)) received a single injection of ACSF (Tocris Bioscience) (0.5 µL) into the AcbSh ipsilateral hemisphere compared to the Es-VTA hemisphere. This injection, administered just before the target Es-VTA, served as a control for the amphetamine injection (D-Amphetamine Sulfate, Sigma-Aldrich, Steinheim, Germany) into the AcbSh (5.0 µg/0.5 µL) for rats used in the behavioral experiment (after ACSF as HR/LR control behavioral groups and after amphetamine injection as HR/LR experimental behavioral groups) (Figure 11(a7,a8)). The brains of these rats were used for neurochemical studies immediately after amphetamine injection (as HR*_Amph_*/LR*_Amph_* experimental groups). In contrast, the brains for the control groups in the neurochemical studies were derived from rats that received only ACSF into the AcbSh before Es-VTA (HR*_ACSF_*/LR*_ACSF_* control groups, Figure 11(b7)).

The behavioral experiment was conducted in a test box (22 cm × 35 cm × 44 cm) placed within a soundproof chamber. The rats, removed from their home cages, were allowed 30 min to explore the test box and habituate to the experimental conditions. To ensure satiation, all animals had ad libitum access to food (Labo feed, Poland) and water during the experiment. Each initial stimulation session consisted of 45 cycles, with each cycle lasting 30 s of stimulation followed by a 20-s rest period. The current intensity (80–260 µA) was individually adjusted based on the iFR latency (maximum of 5 s). The frequency (50 Hz) and pulse width (0.1 ms) remained constant. In the target Es-VTA sessions, the stimulation frequency varied in four blocks of trials (from 81.38 Hz to 17.71 Hz and vice versa). The iFR reaction latency (20 s) was determined by linear interpolation for the iFR latency-frequency function. All details regarding feeding and criteria for defining the response have been included to ensure the reproducibility of results.

All injections were performed with a 10 µL Hamilton syringe (0.28 mm diameter) and an injection pump (Kd Scientific; RoHS Compliant, Holliston, MA, USA) (injection volume 0.5 µL). Each electrical stimulation session used a stimulator (Hugo Sachs Elektronik Type 215, Harvard Apparatus GmbH, March-Hugstetten, Germany), and the current parameters were monitored with an oscilloscope (GOS-620 GW Instek, TME Electronic Components; Łódź, Poland). Additionally, all stimulation sessions (initial and target Es-VTA) were recorded using two cameras (BuyBest Easy Snap HD, Richfield, MN, USA), and a computer analyzed behavior.

To summarize, rats that received ACSF and amphetamine injections into the AcbSh were used exclusively for behavioral studies (Figure 11(a7,a8)). Their brains were later utilized in neurochemical studies as experimental groups. The brain rats that received only ACSF injections into the AcbSh were exclusively control groups for neurochemical studies (Figure 11b).

### 4.4. Histological and Immunofluorescence Procedures

Before staining, all animals were sacrificed two hours after completing the behavioral procedure (pentobarbital, 0.9% saline, and 4% phosphate-formaldehyde buffer). During histological procedures, Nissl staining was used to identify the placement of the stimulating electrode in the VTA and the injection cannula in the AcbSh. Selected sections were prepared from 4.52 to 5.80 mm caudal to bregma and 2.28 to 1.08 mm rostral to bregma, with each section having a thickness of 20 μm (Leica CM 1850, Leica Biosystems, Deer Park, TX, USA). These sections were mounted on gelatin-coated glass slides (Superfrost™ Plus Adhesion Microscope Slides with Tab; Epredia, Netherlands B.V., Breda, The Netherlands) treated with Chromium (III) and Potassium Sulfate (Merck KGaA, Darmstadt, Germany). After drying, they were stained with cresyl violet perchlorate (Sigma-Aldrich, St. Louis, MO, USA), dehydrated, and coverslipped with DPX mounting medium for histology (Sigma-Aldrich, St. Louis, MO, USA). Micrographs of histological sections were taken simultaneously using templates derived from the rat brain atlas [42], using a magnifier (Stemi 508; Zeiss, Oberkochen, Germany) and a camera (Axiocam 105 color; Zeiss, Oberkochen, Germany) with integrated software (Zen Digital Imaging; Zeiss, Oberkochen, Germany).

Immunofluorescence procedures were then performed on the remaining sections to image *c-fos* expression (density of cFos+ cells), DAPI, and cells expressing tyrosine hydroxylase (TH+) and choline acetyltransferase (ChAT+) activity.

All steps of the triple staining procedure were performed at room temperature, using phosphate-buffered saline (PBS; pH 7.4), normal goat serum (NGS; Sigma-Aldrich, St. Louis, MO, USA; 5% NGS containing 0.3% Triton X-100), 0.5% bovine serum albumin (BSA; Sigma-Aldrich, St. Louis, MO, USA). The first step of immunofluorescence staining was incubation for 48 h with a solution containing primary antibodies: *c-fos* (monoclonal mouse cFos Antibody, Santa Cruz Biotechnology, Dallas, TX, USA; dilution 1:200), TH+ (polyclonal rabbit anti-TH, Merck KGaA, Darmstadt, Germany or monoclonal mouse Anti-TH, Novus Biologicals, Centennial, CO, USA; dilution 1:1500) and ChAT+ (monoclonal mouse ChAT Antibody, Novus Biologicals, Centennial, CO, USA; dilution 1:500) in PBS containing 0.3% TritonX 100 and 3%NGS, then washed with PBS-Tris hydrochloride (Tris pH 7.0–9.0, Merck KGaA, Darmstadt, Germany) and incubated for 4 h with CF488 Goat Anti-Rabbit IgG, spectrally similar to Alexa Fluor 488/CF543 Goat Anti Mouse IgG, spectrally similar to Alexa Fluor 546; Invitrogen, Waltham, MA, USA; dilution of 1:500).

The staining process was completed by embedding using a hardening resin combined with DAPI (EverBrite Hardset Mounting Medium with DAPI; Biotium, Fremont, CA, USA) and by applying coverslips (24 × 60 mm, Bionovo, Legnica, Poland). Fluorescence images were taken using a PrimoStar microscope (three-channel system) (Carl Zeiss MicroImaging GmbH, Göttingen, Germany) (magnification 20 × 10). All photos were labeled with a white label on a black background, with a grayscale from 0 (black) to 255 (white), and processed using Carl Zeiss Imaging Systems software (Oberkochen, Germany, Axio Vision Rel. 4.9.1). The boundaries of brain structures were determined based on the rat brain atlas [42] using contour traces from the templates. The software counted a defined area of brain structures (i.e., thresholding procedure), where optical density and size filters were calibrated to count white grains (>70% white) exceeding 15 pixels (15 μm^2^). Thus, the software excluded any remaining particles inconsistent with the size of the cell nucleus (cFos/DAPI) or cells containing TH+/ChAT+ at a given magnification (20 × 10). The density of cFos-positive cells (cFos+), DAPI (DAPI+) as the number of counted nuclei, and TH (TH+)/ChAT (ChAT+) cells per 1 mm^2^ of the surface area of the analyzed structure were then counted. Microscopic analysis of cFos protein was performed in selected forebrain and midbrain structures, TH+ cells in the dopaminergic A8–A15 cell groups, and ChAT+ cells in the cholinergic Ch1–Ch6 cell groups.

### 4.5. Data Analysis

The results were meticulously analyzed, ensuring a thorough understanding of the data, and presented as line and bars (mean + SE) and box-and-whisker plots representing median values, first and third quartiles, and maximum/minimum values collapsed over the four anteriority levels; all data analyses were performed using SPSS 17.0 and Gretl software (1.9.4 version). Before statistical analysis, tests were conducted to determine the normality of the distribution and equality of variances, ensuring the precision of our findings. The data presented in this study did not achieve compliance with a normal distribution.

Our study meticulously examined the correlation between the percentage change in the frequency threshold for iFR by ES-VTA immediately following amphetamine administration to the AcbSh and the locomotor activity score in the novelty test. This analysis was conducted for two distinct behavioral groups: HR*_Amph_* (n = 5) and LR*_Amph_* (n = 5). Regression analysis was performed using the Spearman correlation coefficient (Rs), with statistical significance set at *p* < 0.05, ensuring the reliability and precision of our findings. In addition, percent changes in the threshold after amphetamine injection, calculated based on the control (baseline as ACSF injection), and differences during the iRF-latency(s) were analyzed (Mann–Whitney U test). Moreover, this latency reaction(s) (after amphetamine injection) changes were precisely compared with the latency of behavioral reaction pre-injection baseline and latency reaction after ACSF injection as a control (Wilcoxon’s signed rank test).

The Wilcoxon signed-rank test was also used to analyze immunofluorescent staining (TH+ and ChAT+ cells and cFos+ nuclei). The analysis, thoroughly conducted to detail, included not only immunofluorescent staining of the nervous tissue of experimental animals (HR*_Amph_*, n = 5; LR*_Amph_*, n = 5) (Figure 11a) but also for the experimental animals, explicitly prepared separate control animals—10 rats after the novelty test, ACSF injection into the AcbSh, and electrical stimulation of the VTA (HR*_ACSF_*, n = 5; LR*_ACSF_*, n = 5) (Figure 11b). Therefore, comparisons between both experimental and control groups (HR*_ACSF_* vs. HR*_Amph_*; LR*_ACSF_* vs. LR*_Amph_*; HR*_ACSF_* vs. LR*_Amph_*; and LR*_ACSF_* vs. HR*_Amph_*) and within them (HR*_ACSF_* vs. LR*_ACSF_*; HR*_Amph_* vs. LR*_Amph_*) were assessed. In the case of TH+ cells, dopaminergic cells A8–A15 were analyzed, with particular attention paid to the division into structures in the case of dopaminergic cells A10 and A9. Analysis of CHAT+ cells included groups of Ch1–Ch6 cells. In the case of Fos protein, the study included selected forebrain and midbrain structures.

The *p*-value lower than 0.05 was considered statistically significant.

## Data Availability

The data presented in this study are available upon request from the corresponding author.

## Abbreviations

A15–A18	dopaminergic cell groups
AADC	dopamine decarboxylase enzyme
ACo	antero-cortical nucleus of the amygdala
ACSF	artificial cerebrospinal fluid
*Acsf*	control groups with ACSF injection
AD	anterodorsal thalamic nucleus
AH	anterior hypothalamic area
AM	anteromedial thalamic nucleus
*Amph*	experimental groups with amphetamine injection
Arc	arcuate hypothalamic nucleus
AV	anteroventral thalamic nucleus
Acb	accumbens nucleus
AcbC	accumbens nucleus, part core
AcbSh	accumbens nucleus, part shell
BLA	basolateral nucleus of the amygdala
BST	bed nucleus of the stria terminalis
CA1–CA3	hippocampal fields
cAMP	cyclic adenosine monophosphate
CDK5	cyclic-dependent kinase
Ce	central nucleus of the amygdala
Ch1–Ch6	cholinergic cell groups
CG1	cingulate cortex, area 1
CG2	cingulate cortex, area 2
ChAT	choline acetyltransferase
CL	mediolateral thalamic nucleus
Cli	caudal linear nucleus of the raphe
CM	centralo-medial thalamic nucleus
CNS	central nervous system
Cpu	caudate putamen
DA	dorsal hypothalamic nucleus
DAT	dopamine transporter protein
DG	dentate gyrus
DMD	dorsomedial hypothalamic nucleus
Es-VTA	electrical VTA-stimulation
iFR	induced feeding response
IF	interfascicular nucleus
GP	globus pallidum
GR	glucocorticoid receptors
HDB	nucleus of the horizontal limb of the diagonal band
HR	rats with high locomotor response to novelty test
IAM	inter anteromedial thalamic nucleus
IMD	inter mediodorsal thalamic nucleus
LDT	laterodorsal tegmental nucleus
LH	lateral hypothalamic area
LHb	lateral habenular nucleus
LR	rats with low locomotor response to novelty
LSD	lateral septal nucleus, dorsal part
LSI	lateral septal nucleus, intermediate part
LSV	lateral septal nucleus, ventral part
MD	mediodorsal thalamic nucleus
Me	medial nucleus of the amygdala
MHb	medial habenular nucleus
ML	mesolimbic system
mPFC	medial prefrontal cortex
MS	medial septal nucleus
PAG	periaqueductal gray
PBP	parabrachial pigmented nucleus of the VTA
PC	paracentral thalamic nucleus
PFC	prefrontal cortex
PH	posterior hypothalamus
PN	paranigral nucleus of the VTA
PPN	pedunculopontine tegmental nucleus
PRh	perirhinal cortex
PT	paratential thalamic nucleus
PVA	paraventricular thalamic nucleus
PVN	paraventricular hypothalamic nucleus
Re	reuniens nucleus
Rh	rhomboid nucleus
Rli	rostral linear nucleus of the raphe
RRF	retrorubral field
RSA	retrosplenial agranular cortex
RSG	retrosplenial granular cortex
Rt	reticular nucleus
SN	substantia nigra
SNC	substantia nigra, compact part
SNL	substantia nigra, lateral part
SNR	substantia nigra, reticular part
SO	supraoptic nucleus of the hypothalamus
SP-MF	moss fibers of the CA3 pyramidal cell
TH	tyrosine hydroxylase
VDB	nucleus of the vertical limb of the diagonal band
vMAT2	vesicular monoamine transporter
VMH	ventromedial hypothalamic nucleus
VP	ventral pallidum
VTA	ventral tegmental area
ZI	zona incerta
